# Statistical misreasoning in online content about vaccines: Implications and recommendations for addressing disinformation

**DOI:** 10.1371/journal.pone.0355341

**Published:** 2026-08-03

**Authors:** Michal Ordak

**Affiliations:** 1 Centre of Regenerative Medicine, Medical University of Bialystok, Bialystok, Poland; 2 Department of Pharmacotherapy and Pharmaceutical Care, Faculty of Pharmacy, Medical University of Warsaw, Warsaw, Poland; Universidad de Granada, SPAIN

## Abstract

**Background:**

Statistical misreasoning is a key mechanism through which anti-vaccine narratives distort scientific information and undermine public confidence in immunisation. Although prior research has examined thematic and ideological features of vaccine misinformation, little is known about the specific errors in numerical reasoning that shape users’ interpretations of vaccine-related data.

**Methods:**

A total of 597 Polish-language Facebook posts expressing anti-vaccine views and containing references to statistical information were analysed. Based on previous research on statistical cognition and an inductive review of the material, a coding scheme comprising ten categories of statistical misreasoning was developed and applied to all posts. Quantitative analyses were then conducted to examine how frequently these categories occurred and which combinations of errors appeared together.

**Results:**

The most prevalent forms of misreasoning were the correlation–causation fallacy (70%, p < 0.001) and base rate neglect (58%, p < 0.001). Denominator neglect and cherry picking appeared in half of the posts, while the remaining categories were less frequent. Most posts contained multiple errors (median = 4), and the most common configuration involved the correlation–causation fallacy, base rate neglect and denominator neglect. The distribution of error counts further showed that posts most often exhibited four distinct categories of misreasoning (23%), followed by three (19%) and five (17%), and overall a majority of posts (62%, p < 0.001) contained between one and four different types of errors. Co-occurrence analysis revealed stable structural patterns, with the strongest association observed between denominator neglect and intuitive reasoning error (ϕ = 0.23; p < 0.001).

**Conclusion:**

Anti-vaccine discourse exhibits systematic patterns of statistical misreasoning that shape erroneous interpretations of vaccine-related data, highlighting the need to address cognitive and statistical misunderstandings through targeted public health communication.

## Introduction

Online environments can enable misleading vaccine narratives to circulate widely, shaping public perceptions and weakening trust in scientific institutions. Attempts to restrict such content on major platforms illustrate the difficulty of limiting misinformation within systems that offer users multiple pathways for sharing and amplifying it. These structural features can allow misleading material to persist and evolve despite formal moderation efforts, sustaining its visibility and influence [1]. False information about vaccines can spread rapidly and reach wide audiences, often more effectively than accurate scientific messages. Such content can weaken confidence in immunization and reduce trust in recommendations from health authorities. Its influence is reinforced by recurring argumentative patterns that rely on distorted evidence and flawed reasoning. Understanding how these patterns function is essential, as vaccine-related misinformation continues to intensify online [2].

Recent research has documented how misinformation about vaccines circulates across multiple social media platforms and how different themes shape public perception. For example, a systematic review of 45 empirical studies examining COVID-19 vaccine misinformation on social media found that false claims circulated across multiple platforms and were dominated by themes related to medical misinformation, vaccine development, and conspiracy narratives. The included studies, most of which originated from Western countries, showed that Twitter and Facebook were the primary channels through which misleading vaccine content spread. Evidence from 19 of these studies indicated that exposure to such misinformation was consistently associated with increased vaccine hesitancy or reduced uptake [3]. An examination of 4511 UK vaccine-related tweets posted in 2019 identified 334 messages opposing vaccination, generated by users who were generally active and well connected in the platform’s interaction network. These anti-vaccine posts predominantly framed vaccination as harmful through personal accounts and value-based reasoning, and although anonymity did not alter the thematic content, it was associated with producing a greater volume of such messages [4]. Across the five national samples from the United Kingdom, Ireland, the United States, Spain and Mexico, a substantial number of respondents regarded widely circulated COVID-19 misinformation as highly credible. Greater susceptibility to these misleading claims was associated with poorer compliance with public health recommendations and reduced willingness both to be vaccinated and to encourage vaccination among vulnerable individuals [5]. Previous studies analyzing social media posts suggest that antivaccine misinformation often relies on recurring thematic patterns and specific stylistic choices that shape how such content circulates online. Evidence from an examination of 140 Facebook posts indicates that safety-related narratives dominate these messages, while strategies such as conversational framing or imitation of news and scientific formats contribute to their visibility and engagement [6]. Another study examining Facebook discussions in Poland identified a wide range of recurring arguments used to oppose COVID-19 vaccines, revealing twelve distinct categories that included both long-standing antivaccine themes and several narratives specific to the pandemic [7]. Research has shown that online opposition to vaccination can intensify rapidly during major public health events, accompanied by shifts in the themes that dominate discussion. Analyses of Twitter activity during the emergence of COVID-19 indicate substantial growth in antivaccine narratives and increasing mistrust directed at health authorities, highlighting the scale at which online discourse can shape vaccine attitudes [8]. Antivaccine discourse on Facebook has increasingly adopted a civil-liberties and politically oriented frame, with previously distinct strands of opposition converging into a more uniform narrative that positions vaccine refusal as a matter of personal rights [9]. One review concerning vaccination attitudes in Asia showed that social media platforms played a substantial role in shaping both COVID-19 and routine immunization perceptions during the peri-pandemic period. The evidence indicated that reliance on these platforms was linked to greater exposure to misinformation and conspiracy narratives, which in turn heightened vaccine hesitancy and delayed childhood immunizations [10].

Accurate interpretation of published findings can strengthen trust in medicine, whereas errors in statistical reasoning foster growing doubt and susceptibility to disinformation. Despite the increasing number of studies on health-related disinformation, there is still a lack of published articles showing how individuals with anti-vaccination views arrive at their beliefs through flawed statistical reasoning. Existing work focuses primarily on the narrative and ideological aspects of anti-vaccination content, leaving the interpretative processes that lead users to form specific judgments about vaccination poorly understood. This gap highlights the need to examine not only what anti-vaccine messages claim, but how individuals interpret data and construct erroneous conclusions. For this reason, the aim of this manuscript is to identify and describe forms of statistical misreasoning that appear in anti-vaccination content and shape erroneous beliefs about vaccines. This approach makes it possible to capture how distorted interpretations of data, relationships, and risk become the basis of narratives that undermine confidence in immunization. This analysis enables the identification of dominant reasoning patterns present in posts opposing vaccination. An additional aim is to outline practical recommendations for reducing statistical misreasoning among individuals who form their views on vaccination based on incorrect interpretations of data.

## Materials and methods

### Data collection and sample selection

Between January 2023 and December 2025, a total of 597 Facebook posts were collected from Polish-language online spaces discussing opposition to vaccination or criticizing immunization policies. The exact sources are not disclosed to protect the confidentiality of online communities and to avoid directing attention to specific discussions. Posts were identified through purposive sampling from publicly accessible Facebook spaces and were screened systematically using predefined inclusion and exclusion criteria, rather than being selected in an ad hoc manner. The posts were accessible to the researcher at the time of data collection. Posts were selected using criteria tailored to the aims of this study. The analysed material consisted of original user-generated posts in which individuals expressed their own interpretations of numerical or statistical information related to vaccination. The sample was intentionally restricted to posts expressing opposition to vaccination or scepticism toward immunisation, with a particular focus on posts in which users actively interpreted numerical or statistical information to support such views. These posts included both standalone statements and posts engaging with external content, such as news articles, provided that users offered their own interpretation of the data. Posts that merely reproduced or shared external information without additional interpretation were excluded from the analysis. Only posts in which users referred to numerical information, statistical data, scientific evidence, epidemiological figures, comparisons between groups, or claims involving frequencies, risks, or probabilities concerning various vaccines were included. The focus was therefore not on general anti-vaccination sentiment, but on instances where users attempted to interpret or present data in support of their views. Posts that consisted solely of emotional statements, political commentary without reference to data, memes without text, link-sharing without interpretation, or off-topic content were excluded. Posts were identified through manual searches of publicly accessible Facebook content using relevant keywords and thematic exploration of pages and groups. Only publicly available data were collected, and no private or restricted content was accessed. The data collection and analysis complied with the terms and conditions of the Facebook platform.

### Ethical consideration

As the study involved only publicly accessible online content and collected no personal or identifiable information about users, formal ethics approval was not required. According to the institutional guidelines, research involving analysis of publicly accessible, non-identifiable social media content does not require approval from a bioethics committee. The Bioethics Committee of the Medical University of Warsaw acknowledged the study and confirmed that no formal review was required.

### Statistical misreasoning categories in posts

The coding scheme used in this study was developed to identify distinct forms of statistical misreasoning present in anti-vaccination discourse. The aim was to construct a set of categories that would capture the full range of erroneous interpretations of numerical information observed in the posts while maintaining conceptual clarity and minimizing unnecessary conceptual redundancy. To achieve this, the development of the coding framework drew on established research describing common mistakes people make when interpreting numbers and statistical information, together with an inductive review of the collected posts. This process resulted in the identification of ten categories, each representing a theoretically grounded and empirically observable pattern of misinterpretation.

The selection of categories was informed first by the author’s extensive experience as a statistical editor for multiple academic journals, and second by research documenting key sources of numerical misunderstanding, including limitations of correlation-based inference [11], underweighting of base-rate information [12], difficulties arising from insufficient attention to denominators [13], biased information sampling aligned with prior beliefs [14], differences in how risk formats shape interpretation [15], intuitive pattern-seeking and rapid coherence building in uncertainty [16], overestimation of verbally conveyed probabilities [17], and the effects of misleading or suboptimal visualizations on comprehension [18]. Because statistical reasoning errors tended to appear together rather than as separate phenomena, a synthetic example illustrating the most frequent pattern is provided here. Accordingly, the categories were not treated as fully mutually exclusive. Individual posts frequently contained multiple overlapping forms of statistical misreasoning, and the coding framework was intended to identify analytically distinguishable patterns of reasoning rather than fully discrete error types. The most common co-occurrence involved the correlation–causation fallacy, base rate neglect, and denominator neglect. For instance, a user might state: “I won’t take the vaccine because most of the patients currently in intensive care are vaccinated, which shows that the vaccine must be causing severe illness. If vaccinated people make up the majority of hospital cases, it is clear evidence that the risks outweigh the benefits.” This type of reasoning simultaneously treats correlation as causal evidence, ignores the underlying population proportions, and focuses solely on absolute case counts without considering denominators.

The first category, confusion of correlation and causation, refers to interpreting two temporally or coincidentally related events as evidence of a causal link. Base rate neglect captures instances where users disregard the underlying prevalence of vaccinated and unvaccinated individuals when drawing conclusions from numerical comparisons. Denominator neglect refers to interpreting absolute numbers without reference to the size of the population from which they derive. Cherry picking describes the selective use of numerical evidence such as isolated datasets, individual countries, or short time windows to support a predetermined conclusion. Misunderstanding the distinction between relative and absolute risk occurs when proportional changes are interpreted without consideration of the baseline values from which they arise. The small sample fallacy involves drawing general conclusions from very small sets of observations, often anecdotal in nature. Intuitive statistical reasoning describes cases in which individuals replace complex probabilistic judgments with simplified evaluative heuristics. Misinterpretation of random fluctuations refers to inferring meaningful trends from noise or normal variation in data. Overinterpretation of percentages without reference to base values reflects the use of percentage changes in the absence of context necessary for their proper interpretation. Finally, misreading graphical scales concerns errors arising from truncated axes, altered proportions, or other visual manipulations that distort numerical relationships. Coding decisions were based on identifying patterns of reasoning in the posts that matched the conceptual definitions of each category. Each category was applied using consistent criteria derived from its definition, focusing on how numerical information was interpreted within the post. The coding scheme was applied systematically across the entire dataset. The final number of categories was not predetermined but emerged from an iterative process of refining and grouping observed patterns of misreasoning. The process involved initial identification of distinct instances of misreasoning in the posts, followed by iterative grouping and refinement of these instances into broader categories based on conceptual similarity. Fewer categories resulted in a loss of important conceptual distinctions between different types of errors, whereas introducing additional categories led to conceptual overlap without adding analytical value. The set of ten categories therefore reflects a balance between conceptual completeness and analytical distinctiveness. These ten categories were selected because they collectively provide a comprehensive yet analytically differentiated typology of the statistical reasoning errors observed in the posts. Using fewer categories would hide important differences between types of errors. For example, base rate neglect and denominator neglect look similar on the surface but are actually caused by different ways of thinking. On the other hand, adding more than ten categories would make some of them overlap with each other, which would make the coding less clear and less reliable without improving the analysis. The final framework therefore represents a balanced structure: broad enough to encompass all identifiable forms of statistical misinterpretation in the material, yet specific enough to allow for consistent classification and clear analytical interpretation.

In Table 1, the ten categories of statistical misreasoning are presented, together with their definitions and illustrative examples.

**Table 1:** Categories of statistical misreasoning identified in the analyzed posts, with definitions and illustrative examples.

Category	Definition	Illustrative example
Correlation–causation fallacy	Interpreting two temporally or coincidentally related events as evidence of a causal link.	“Some people developed symptoms shortly after vaccination, which means the vaccine must be the cause of these symptoms.”
Base rate neglect	Disregarding the underlying prevalence of vaccinated and unvaccinated individuals when drawing conclusions from numerical comparisons	“Among hospitalized patients, vaccinated individuals are more numerous than unvaccinated ones, which shows the vaccine is ineffective.”
Denominator neglect	Interpreting absolute numbers without reference to the size of the population from which they are derived	“There were 200 cases of complications among vaccinated people last month, which is a very high number.”
Cherry picking	Selective use of numerical evidence (e.g., specific datasets, countries, or time periods) to support a predetermined conclusion	“In one selected country, vaccination rates increased while case numbers also increased, which proves that vaccines are not effective.”
Relative vs absolute risk error	Interpreting proportional changes without consideration of the baseline values from which they arise	“The risk increased by 100% after vaccination, so the increase is substantial.”
Small sample fallacy	Drawing general conclusions from very small sets of observations, often anecdotal in nature	“A few people I know experienced adverse reactions after vaccination, so it is clear that the vaccine is unsafe.”
Intuitive reasoning error	Replacing complex probabilistic judgments with simplified evaluative heuristics	“It does not seem plausible that a vaccine developed so quickly could be safe, so I do not trust it.”
Random fluctuation misinterpretation	Inferring meaningful trends from noise or normal variation in data	“The number of cases rose over the past few days, which shows that the vaccination program is not working.”
Overinterpretation of percentages	Using percentage changes in the absence of the base values necessary for their proper interpretation	“Adverse events increased by 50% after vaccination, which indicates a serious problem.”
Graphical scale misreading	Misinterpreting numerical relationships due to distorted or misleading graphical scales (e.g., truncated axes)	“The graph shows a sharp increase in deaths after vaccination, so the trend is clearly rising.”

### Statistical analysis

Statistical analysis was conducted using the IBM SPSS Statistics 25 package. The analysis proceeded in several steps reflecting the aims of the study. First, descriptive statistics were used to determine the prevalence of each statistical misreasoning category across all posts. For every category, the proportion of posts in which the error occurred was calculated, and chi-square tests were applied to assess whether the occurrence of the most frequent errors exceeded 50% of the sample, which was treated as an indicator of majority occurrence and used to identify dominant patterns of misreasoning. Second, to examine the complexity of statistical misreasoning within individual posts, the number of categories assigned to each post was computed, and the distribution of category counts was summarised using mean, median, minimum and maximum values, as well as frequency proportions for each count level. Third, to identify the most common configurations of misreasoning, co-occurrence patterns were analysed by calculating all unique three-, four-, and five-category combinations, after which the five most frequent combinations at each level were selected for reporting. Finally, to assess the internal coherence of the dominant multi-category patterns, pairwise associations between categories were analysed using the phi coefficient. Phi values were computed for all pairs of categories included in the most frequent combinations. Only statistically significant associations, which allowed for the interpretation of the corresponding phi coefficients, were retained for interpretation. The statistically significant level was p < 0.05. The analysis of co-occurrence patterns was exploratory in nature and was not based on pre-specified theory-driven hypotheses. The statistical analysis was primarily descriptive and aimed at identifying dominant patterns and structural relationships rather than conducting exhaustive comparative testing between categories.

## Results

### Prevalence of statistical misreasoning in analysed posts

A total of 597 posts that contained users’ interpretations of statistical information concerning various vaccines were included in the analysis (Table 2). Across the entire sample, the most prevalent form of statistical misreasoning was the correlation–causation fallacy, identified in 420 posts (70%). A similarly high proportion of posts exhibited base rate neglect, which appeared in 347 cases (58%). For both of these categories, the proportion of posts in which the error occurred exceeded 50% of the sample (p < 0.001), indicating that these forms of misreasoning were present in the majority of posts and represented dominant patterns in antivaccination discourse. The 50% threshold was treated as a pragmatic indicator of majority occurrence within the analysed sample and was used to distinguish dominant from less prevalent forms of statistical misreasoning. Two additional categories, denominator neglect and cherry picking, appeared in approximately half of all posts (296 posts, 50%, and 295 posts, 49%, respectively). The remaining categories occurred in a smaller portion of the posts. Among them, the most frequent was the small sample fallacy, identified in 263 posts (44%). Intuitive reasoning error was also relatively common (238 posts, 39.9%), followed by overinterpretation of percentages (189 posts, 32%) and relative vs absolute risk error (160 posts, 27%). Less frequent were misinterpretation of random fluctuations (127 posts, 21%) and misreading graphical scales (76 posts, 13%), which appeared in only a minority of the examined content.

**Table 2:** Prevalence of statistical misreasoning categories across 597 analysed posts.

Category	n	%	Statistical test result*
1. Correlation–causation fallacy	420	70	χ^2^(1) = 98.91; p < 0.001
2. Base rate neglect	347	58	χ^2^(1) = 15.76; p < 0.001
3. Denominator neglect	296	50	χ^2^(1) = 0.04; p = 0.84
4. Cherry picking	295	50	χ^2^(1) = 0.08; p = 0.78
6. Small sample fallacy	263	44	χ^2^(1) = 8.44; p = 0.004
7. Intuitive reasoning error	238	40	χ^2^(1) = 24.52; p < 0.001
9. Overinterpretation of percentages	189	32	χ^2^(1) = 80.34; p < 0.001
5. Relative vs absolute risk error	160	27	χ^2^(1) = 128.52; p < 0.001
8. Random fluctuation misinterpretation	127	21	χ^2^(1) = 197.08; p < 0.001
10. Graphical scale misreading	76	13	χ^2^(1) = 331.7; p < 0.001

*chi-square test

### Number of categories assigned per post

**Fig 1:**
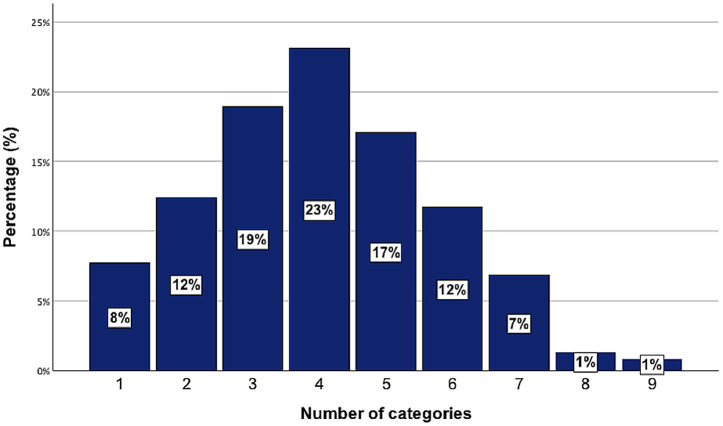
Distribution of the number of categories assigned per post.

The median and mean number of categories assigned per post were both 4, with a minimum of 1 and a maximum of 9. Posts were most commonly assigned four categories (23%), followed by three categories (19%) and five categories (17%). The majority of posts, n = 371 (62%; p < 0.001), were assigned between one and four categories (Fig 1).

### Co-occurrence patterns of statistical misreasoning categories

Analysis of the most frequent co-occurring categories showed that statistical misreasoning appeared in clear and recurrent configurations (Table 3). The most common triplet consisted of the correlation–causation fallacy, base rate neglect and denominator neglect, which co-occurred in 139 posts. This core triplet was present in three of the five most frequent quadruplets and in three of the five most frequent quintuplets, indicating that these three errors often form the backbone of more complex misinterpretations. Additional errors most frequently extending this core were small sample fallacy, cherry picking and intuitive reasoning error. These categories appeared repeatedly in the most frequent four- and five-element combinations, suggesting that they serve as typical extensions of the basic misreasoning pattern. The most common quadruplet combined the core triplet with intuitive reasoning error, occurring in 83 posts, while other frequent quadruplets added cherry picking or small sample fallacy in various configurations. Quintuplets showed similar regularities: the two most frequent five-category combinations included the core triplet together with either cherry picking or small sample fallacy and intuitive reasoning error, and the remaining quintuplets were also composed of the same restricted set of errors, sometimes omitting one of the core categories. Although five-category combinations were less common overall than triplets and quadruplets, their structure closely mirrored the patterns observed in smaller combinations. Taken together, these results indicate that statistical misreasoning in antivaccine posts is organised around a small set of recurring core errors that are systematically expanded by a limited number of additional interpretative distortions.

**Table 3:** Most frequent triplet, quadruplet and quintuplet combinations of statistical misreasoning categories.

Combination size	Categories	Count
Triplet	Correlation–causation fallacy + Base rate neglect + Denominator neglect	139
	Correlation–causation fallacy + Base rate neglect + Small sample fallacy	122
	Correlation–causation fallacy + Denominator neglect + Cherry picking	122
	Correlation–causation fallacy + Base rate neglect + Intuitive reasoning error	120
	Correlation–causation fallacy + Base rate neglect + Cherry picking	117
Quadruplet	Correlation–causation fallacy + Base rate neglect + Denominator neglect + Intuitive reasoning error	83
	Correlation–causation fallacy + Base rate neglect + Denominator neglect + Cherry picking	76
	Correlation–causation fallacy + Base rate neglect + Cherry picking + Intuitive reasoning error	71
	Correlation–causation fallacy + Denominator neglect + Small sample fallacy + Intuitive reasoning error	70
	Correlation–causation fallacy + Base rate neglect + Denominator neglect + Small sample fallacy	67
Quintuplet	Correlation–causation fallacy + Base rate neglect + Denominator neglect + Cherry picking + Intuitive reasoning error	50
	Correlation–causation fallacy + Base rate neglect + Denominator neglect + Small sample fallacy + Intuitive reasoning error	50
	Correlation–causation fallacy + Denominator neglect + Cherry picking + Small sample fallacy + Intuitive reasoning error	47
	Correlation–causation fallacy + Base rate neglect + Cherry picking + Small sample fallacy + Intuitive reasoning error	43
	Correlation–causation fallacy + Base rate neglect + Denominator neglect + Cherry picking + Small sample fallacy	42

To further examine the internal coherence of the most frequent multi-category patterns, pairwise associations between the statistical misreasoning categories were analysed using the phi coefficient. Phi values were computed for all pairs of categories that appeared within the dominant triplets, quadruplets, and quintuplets identified above. This procedure was not intended to replicate the frequency-based analysis of co-occurrences but to assess whether the categories forming these recurrent combinations also showed measurable pairwise associations across the full dataset. This additional step made it possible to identify which individual pairs of errors formed statistically coherent links within the broader multi-category patterns, thereby clarifying which relationships reflected genuine structural connections rather than mere co-occurrence. In line with this objective, only statistically significant associations were retained for interpretation. Among the examined pairs, five showed notable associations. The strongest relationship was observed between denominator neglect and intuitive reasoning error (ϕ = 0.23, p < 0.001). Additional meaningful links were found for base rate neglect with intuitive reasoning error (ϕ = 0.14, p = 0.001) and for small sample fallacy with intuitive reasoning error (ϕ = 0.13, p = 0.002). Two further pairs demonstrated weaker but statistically reliable associations, namely denominator neglect with cherry picking (ϕ = 0.11, p = 0.01) and small sample fallacy with base rate neglect (ϕ = 0.1, p = 0.01). All remaining phi coefficients were close to zero and non-significant, indicating that they did not contribute to the structural configuration of the categories. These results show that, although the most frequent combinations involved multiple categories simultaneously, a subset of dyadic associations, particularly those involving intuitive reasoning error and denominator neglect, also emerged as statistically non-random within the dataset.

## Discussion

### Interpretation of findings in the context of existing research

According to available knowledge, this is the first study to identify and describe forms of statistical misreasoning in anti-vaccine content. Previous analyses of vaccine-related misinformation have focused primarily on thematic narratives and ideological motives, overlooking the interpretative processes underlying the formation of false conclusions based on data. The findings of this study offer a new perspective, demonstrating that anti-vaccine discourse is characterised by recurring patterns of errors in reasoning about numbers, risk, and statistical relationships. In contrast to earlier work, this analysis provides the first comprehensive typology of such errors and reveals their structural configurations in anti-vaccine discussions. In doing so, the study broadens the current understanding of misinformation mechanisms, emphasising the importance of examining how numerical information is processed rather than focusing solely on narrative content.

Confusion of correlation and causation emerged as the most prevalent form of misreasoning, underscoring the central role of erroneous causal inference in the interpretation of vaccine-related statistical information. This finding aligns with prior work showing that difficulties in distinguishing associative from causal relationships are widespread and can persist even in educational contexts explicitly devoted to teaching this distinction [19]. Even when associations are described using strictly non-causal language, people frequently interpret them as evidence of causation, indicating that even minimal correlational phrasing can give rise to unwarranted causal inferences [20]. Base rate neglect constituted the second most frequent error, consistent with evidence showing that individuals often prioritise salient diagnostic information over underlying population proportions, even when additional time or cognitive resources would allow for more balanced consideration of base rates [21]. Denominator neglect emerged as another frequently identified error, reflecting the tendency to focus primarily on absolute event counts while overlooking the size of the populations from which these counts are drawn. This pattern is consistent with evidence showing that individuals often attend disproportionately to numerators and insufficiently to denominators when evaluating treatment risks or risk reductions [22]. Cherry picking also appeared in approximately half of the analysed posts, reflecting the frequent use of selectively chosen sources or examples to support predetermined conclusions. This pattern is consistent with observations that misinformation often relies on marginal or non-mainstream voices presented as authoritative in order to create a false impression of controversy or heightened risk [23]. The small sample fallacy was also frequently observed, aligning with critiques of vaccine-related claims that rely on statistically inadequate sample sizes, as highlighted in analyses demonstrating how undersized datasets can generate misleading or spurious associations [24]. Intuitive reasoning error was also frequently observed, reflecting a reliance on rapid, heuristic judgments in place of systematic evaluation of numerical information. This tendency corresponds with evidence that individuals endorsing anti-vaccination views often exhibit reduced engagement in analytical reasoning, indicating that intuitive, non-evidence-based cognitive processes contribute to the formation of such misinterpretations [25]. The less frequent categories included overinterpretation of percentages, errors in interpreting relative versus absolute risk, misinterpretation of random fluctuations, and misreading of graphical scales. In the case of errors involving relative and absolute risk, prior analyses have demonstrated that reporting vaccine efficacy in terms of relative risk reduction can be inherently misleading and may contribute to disinformation, whereas absolute measures of risk provide a more accurate and informative representation of the true magnitude of effect [26]. These findings can be further situated within a broader body of research on cognitive biases and decision-making. In clinical contexts, cognitive biases have been shown to contribute to diagnostic errors and suboptimal judgments, while structured reasoning approaches may help mitigate their effects and improve decision accuracy [27]. Evidence from surgical research indicates that cognitive biases such as overconfidence, anchoring, and confirmation bias are widespread and can negatively influence performance and patient outcomes across multiple stages of care [28]. Additionally, research in emergency clinical settings demonstrates that a wide range of cognitive biases are present among healthcare professionals, although their precise impact on decisions and outcomes remains complex and not fully resolved [29]. Taken together, this body of work indicates that cognitive biases represent a general and well-established class of systematic deviations in reasoning, which can influence how numerical and risk-related information is interpreted across different domains.

### Structural patterns of misreasoning and underlying cognitive mechanisms

Statistical misinterpretations typically did not occur separately but appeared together within the same message. The most frequent combinations involved correlation–causation fallacy occurring together with base rate neglect and denominator neglect, as well as configurations additionally including small sample fallacy or cherry picking. These patterns indicate that vaccine-related misinterpretations are not based on isolated reasoning failures but arise through the simultaneous use of several interconnected forms of statistical misjudgment. The repeated appearance of the same combinations suggests that certain errors tend to co-occur recurrently in anti-vaccine discourse and may reflect common patterns in the interpretation of numerical information. To explore the coherence of these recurring error configurations, pairwise associations between categories were examined. The most prominent of these links connected denominator neglect with intuitive reasoning error, suggesting that difficulties in recognising the relevance of underlying population sizes may be particularly pronounced when users rely on rapid, heuristic evaluations of numerical information. Such interdependencies suggest that some errors may co-occur systematically in anti-vaccine discourse, although this pattern may also reflect the high prevalence and salience of particular forms of statistical misreasoning. Difficulties in processing numerical information observed in these patterns are consistent with evidence that individuals with lower numeracy are more susceptible to overlooking essential statistical features and to relying on simplified cues when interpreting quantitative data [30]. Such tendencies align with broader research showing that limited numerical competence increases vulnerability to ratio bias, denominator neglect, and other distortions arising from intuitive rather than analytical processing of risk-related information [31,32]. Moreover, the prominence of heuristic reasoning in the identified error configurations corresponds with findings that miserly information processing, as indexed by cognitive reflection performance, is associated with a greater likelihood of relying on rapid, insufficiently examined judgments when evaluating statistical content [33]. These dynamics resemble broader patterns observed in research on anti-vaccine communication, which shows that such messages are often presented in emotionally engaging, cognitively easy formats that facilitate heuristic rather than analytical processing of information. The prominence of personal stories, fear-based cues and simplified explanations may increase the likelihood that multiple statistical misinterpretations co-occur within a single message, reinforcing the interpretative shortcuts identified in this study. Moreover, the tendency for users to evaluate vaccine-related content through motivated reasoning further amplifies these effects, promoting selective acceptance of numerically distorted claims that align with pre-existing beliefs [34].

### Recommendations for reducing statistical misreasoning in vaccine-related online discourse

The prevalence of these forms of statistical misreasoning in online vaccine discourse poses a substantial societal risk, as distorted interpretations of numerical evidence can erode trust in public health recommendations and undermine adherence to vaccination programmes. When such errors circulate widely and remain uncorrected, they may contribute to the rapid normalisation of inaccurate beliefs about vaccine safety and effectiveness, particularly among individuals already inclined to rely on intuitive or motivated reasoning. For this reason, it is necessary to outline recommendations aimed at reducing susceptibility to statistical misinterpretation and strengthening the accuracy with which numerical information about vaccines is understood. The following section presents eight recommendations designed to reduce the most commonly observed forms of statistical misreasoning in anti-vaccine discourse. These recommendations were developed on the basis of the empirical findings of this study, specifically the observed frequency and structural patterns of statistical misreasoning across the analysed posts.

#### - Provide statistical information with explicit base rates and denominators.

Public health communication should consistently include contextual population data, such as the proportion of vaccinated and unvaccinated individuals or the size of groups being compared, to counteract base rate neglect and denominator neglect. Presenting data without these reference values facilitates distorted interpretations of relative risk and encourages erroneous causal inferences. Research on risk communication shows that formats which omit base rate information or rely on relative measures are more likely to be misinterpreted, whereas including such contextual data improves the accuracy of probabilistic judgments [35].

#### - Present risk information using formats that support correct numerical interpretation.

Communicators should prioritise absolute values and natural frequencies (e.g., “5 out of 10,000”) rather than percentages or relative risk figures alone. These formats reduce misinterpretations linked to confusion between absolute and relative risk, and they limit overinterpretation of percentage differences, which can otherwise appear disproportionately large or misleading. Empirical evidence supports this approach, showing that information presented in natural frequencies improves both objective accuracy and subjective understanding of probabilistic data compared to alternative formats [36].

#### - Educate audiences on the distinction between correlation and causation.

Since the correlation–causation fallacy was the most prevalent error in the analysed posts, public messaging should incorporate simple explanations and transparent examples illustrating why co-occurrence does not imply causality. Such materials help users recognise when statistical associations are insufficient to infer causal mechanisms, reducing susceptibility to incorrect causal claims about vaccines.

#### - Counter selective evidence use through multi-source and longitudinal comparisons.

To mitigate cherry picking, institutions should highlight trends supported by consistent data across long time spans, multiple countries, and large samples. Demonstrating convergence across diverse datasets reduces the persuasive power of isolated or selectively chosen examples, which commonly underpin misleading anti-vaccine narratives.

#### - Use visualisations that adhere to best practices in statistical clarity.

Figures should employ untruncated axes, proportional scaling, and transparent labelling to prevent misreading graphical scales. Because distorted or poorly designed visuals can exaggerate differences or conceal trends, improving visual clarity directly reduces one of the documented pathways through which numerical information is misinterpreted. Studies comparing different communication formats indicate that visually supported presentations of risk information are easier to understand and less overwhelming than standard text-based descriptions [37].

#### - Encourage analytical rather than intuitive processing of numerical information.

Interventions should prompt users to examine sample sizes, evaluate data sources, and consider the numerical context before forming judgments. This approach directly addresses intuitive reasoning errors and the small sample fallacy, both of which arise when individuals rely on rapid, heuristic interpretation instead of deliberate evaluation of statistical evidence.

#### - Develop automated tools to detect recurrent patterns of statistical misreasoning.

Fact-checking systems and moderation tools can be designed to recognise the recurring patterns of statistical misreasoning identified in this study, such as the frequent combination of correlation–causation fallacy, base rate neglect, and denominator neglect. Detecting these patterns early allows misleading claims to be identified and corrected before they reach a wide audience.

#### - Equip journalists and online communicators with guidance on accurate statistical reporting.

Because journalists, influencers, and science communicators often serve as intermediaries between scientific data and the public, targeted training should focus on sample size interpretation, absolute versus relative measures, and the variability inherent in epidemiological data. Improving statistical literacy among these actors can substantially reduce the amplification of common misinterpretations observed in online vaccine discourse.

In practice, these recommendations can be incorporated into existing public health communication strategies, including official reports, press releases, and digital campaigns. Their implementation does not require entirely new structures but can build on current communication frameworks by adapting how statistical information is presented and explained. Public health institutions, media organisations, and educational platforms can play a key role in integrating these principles into routine communication practices. In addition, collaboration between statisticians, communication experts, and policymakers may help ensure that statistical information is both accurate and accessible to non-expert audiences. Given the exploratory nature of the present analyses, these recommendations should be treated as indicative and would benefit from further empirical testing in intervention-based research.

### Limitations

This study has several limitations that should be acknowledged. First, the analysis was restricted to Polish-language content, which limits the generalisability of the findings to other linguistic and cultural contexts, and future research would benefit from cross-country comparisons to determine whether similar patterns of statistical misreasoning appear elsewhere. Second, the dataset was drawn exclusively from Facebook, and the structure of discourse, affordances, and algorithmic dynamics differ substantially across social media platforms, meaning that the identified patterns may not fully reflect those present in other online environments. Third, the categorisation of statistical misreasoning was based on the researcher’s qualitative judgement, even though it was grounded in established literature and inductive review, and future studies should incorporate multiple independent coders and assess inter-rater reliability to strengthen methodological robustness. In addition, the absence of independent coding prevents formal assessment of the reproducibility and reliability of classification decisions. Consequently, the proposed classification framework should be regarded as exploratory and theory-generating rather than as a fully validated and independently reproducible coding system. The coding outcomes may therefore partly reflect the researcher’s interpretive perspective, including potential confirmation bias. Future studies should further validate the framework through independent coding procedures and formal assessment of inter-rater reliability, which would allow evaluation of the consistency and reproducibility of classification decisions across coders. Another limitation is that the analysis did not control for message length, which may vary substantially across Facebook posts. Longer posts may provide more opportunities for multiple errors to occur, which could influence the observed co-occurrence patterns and the number of errors identified within a single post. As a result, some of the findings may partly reflect variation in message length rather than differences in the structure of statistical misreasoning itself. A further limitation concerns the independence of observations. The analysis treated each post as a separate unit, although multiple posts may have originated from the same individual, including anonymous users whose identities could not be consistently traced. This may have influenced the observed distribution and co-occurrence of statistical misreasoning. Finally, the analysis focused solely on the content of posts without examining the intentions or motivations of their authors, making it impossible to determine whether the identified errors stemmed from genuine misunderstanding, intuitive processing, or deliberate manipulation. These limitations should be taken into account when interpreting the results and highlight several avenues for future research.

## Conclusions

This study demonstrates that statistical misreasoning in anti-vaccine discourse follows identifiable and recurrent patterns. By revealing the structure and prevalence of these errors, the findings highlight the need for targeted interventions that strengthen statistical understanding and reduce susceptibility to numerically distorted claims in online environments.

## Supporting information

S1 File Anonymized coded dataset. (XLSX)
