# Supplementary material for: Statistical misreasoning in online content about vaccines: Implications and recommendations for addressing disinformation
Source: PLoS One. 2026 Aug 3;21(8):e0355341. doi: 10.1371/journal.pone.0355341 (PMC13432100; doi:10.1371/journal.pone.0355341)
Supplement: Attachment — Submitted filename: Point-by-point-response MARCH.2026.docx [file pone.0355341.s003.docx]

**Point-by-point response template**

**Date: 25.03.2026**

**Manuscript Number:** PONE-D-25-65889

**Title of Article:** “Statistical misreasoning in online content about vaccines: implications and recommendations for addressing disinformation”

**Name of the Corresponding Author: Michal Ordak**

**Email Address of the Corresponding Author:** [**michal.ordak@wum.edu.pl**](mailto:michal.ordak@wum.edu.pl)

Dear Prof. Emily Chenette,

Thank you for sending the Reviewer’s comments regarding my paper entitled “Statistical misreasoning in online content about vaccines: implications and recommendations for addressing disinformation”, by M. Ordak (PONE-D-25-65889). I greatly appreciate all valuable suggestions proposed by the Reviewer, which I consider while preparing the revised version of the manuscript.

**Specific Responses**

**Response to Academic Editor:**

**Comment 1:** “The reviewers have identified three major concerns. First, it is unclear how the labelling system used to define the ten statistical errors was developed. Tools of this kind customarily undergo a process of gradual refinement to minimise the risk of distorting the observed reality, or of the number of labels being arbitrary and thus susceptible to selection bias. Such refinement also guards against overlooking conceptual overlap between labels or, conversely, failing to distinguish between instances assigned to the same label. Second, no measures are described to ensure the reliability of the coding system. Qualitative analyses customarily include safeguards against subjectivity in coding. As noted by one reviewer, this requires, at minimum, that several independent coders apply the same instrument to the same pool of instances, with convergence formally assessed and documented. Third, both reviewers flag the presence of overstatements. While they recognise the value of the descriptive analyses provided, they note that certain assertions are not adequately grounded in the evidence presented.”

**Answer 1:** Thank you for your synthesis of the reviewers’ comments and for identifying the three main areas of concern. I would like to indicate that these points have been addressed in the revised manuscript in line with the reviewers’ suggestions. The methods section has been revised to clarify how the categories of statistical misreasoning were developed and applied. The manuscript now also acknowledges that the coding was conducted by a single researcher and discusses this as a limitation, including the potential for subjectivity and confirmation bias. In addition, statements suggesting that statistical misreasoning drives the persistence or formation of inaccurate beliefs have been removed, and the exploratory nature of the analysis has been more clearly emphasised. I hope that these revisions address the concerns raised.

**Comment 2:** “In your Methods section, please include additional information about your dataset and ensure that you have included a statement specifying whether the collection and analysis method complied with the terms and conditions for the source of the data.”

**Answer 2:** The manuscript was revised to include a description of the data collection process and a statement confirming that the collection and analysis complied with the platform’s terms and conditions.

**Response to Reviewer #1:**

**Dear Reviewer nr 1,**

**Comment 1:** “The main problem of this study is that, at least with the information included in the article, it does not seem sufficiently protected against confirmation bias on the part of the researcher. The content and style of the messages are supposed to reveal ten different statistical errors. It is stated that the ten categories of statistical errors were defined a priori. But were the specific elements or the type of language that reveals each of these errors also defined a priori? If not, there is a risk that the data will be interpreted (that is, messages classified as belonging to one category or another) according to the reader’s expectations. In this regard, both the a priori definition of key message characteristics and the use of a classification rubric, as well as the collaboration of several judges, would have been necessary measures to ensure an adequate interpretation of the data. If these measures were not taken in advance, perhaps the dataset can be now examined by a new team of judges by using a pre-defined set of rules or rubric, so that the conclusions are more robust?”

**Answer 1**: Thank you for this insightful and important comment. The concern regarding potential confirmation bias and the transparency of coding decisions is well taken. In response, the methods section has been revised to clarify that coding decisions were based on identifying patterns of reasoning in the posts that corresponded to the conceptual definitions of each category, and that these criteria were applied consistently across the dataset. In addition, the Limitations section has been expanded to explicitly acknowledge that the coding was conducted by a single researcher without independent verification, which may introduce interpretive bias, including confirmation bias, and may affect the reproducibility of classification decisions. I hope that these clarifications address the reviewer’s concerns.

**Comment 2:** “Similarly: it should also be explained in more detail how the categories were chosen. The justification in the article refers to their number (fewer than 10 would imply that the list is not comprehensive, more than 10 would lead to overlap between categories), but ten is still an arbitrary number (why not 9? 11?) and there is no reasoned explanation or theoretical justification for this decision. Surely the author can provide more information in this respect so that the explanation in the revised article is clear.”

**Answer 2:** Thank you for this helpful comment. The point regarding the potential arbitrariness of the number of categories is well taken. In response, the methods section has been revised to clarify that the final number of categories was not predetermined but emerged from an iterative process of refining and grouping observed patterns of statistical misreasoning in the dataset. The revised text now explains that fewer categories led to a loss of important conceptual distinctions between different types of errors, whereas introducing additional categories resulted in conceptual overlap without adding analytical value. The set of ten categories therefore reflects a balance between conceptual completeness and non-redundancy.

**Comment 3:** “The analysis of structural patterns of misreasoning seems potentially useful at a descriptive level, but it has limitations. For example, it is stated (or inferred from the reading of the article) that this analysis may reveal something useful about the structure of misinformation narratives and discourses, or even about human cognition, but in any case this is indirect evidence that can be interpreted in several ways. To begin with, as far as I understand, it is an exploratory analysis for which there is no clear theory-based hypothesis. That is, it is not proposed a priori that there are certain types of errors that have some kind of association and are therefore expected to appear together frequently. If such hypotheses existed, they should be made explicit in the article.”

**Answer 3:** The methods section has been revised to clarify that the co-occurrence analysis is exploratory in nature and not based on pre-specified theory-driven hypotheses.

**Comment 4:** “The analysis of the co-occurrence of different errors within the same message has other limitations, such as not controlling for message length. Since this study is based on Facebook data and not a microblogging network with fixed character limits (e.g., Twitter), I imagine that message length, measured in number of words, will be highly variable. My reasoning is that the longer the message, the greater the probability of combining several errors in a single message, which could better contextualize the results of this analysis, but this possibility has not been explored.”

**Answer 4:** Thank you for this important observation. The limitations section has been expanded to acknowledge that message length was not controlled for and may influence the number and co-occurrence of errors identified within individual posts.

**Comment 5:** “In general, I believe that important information is missing to understand how the research was conducted. It has already been mentioned that there are no references to any system or rubric for classifying messages. Nor is message length mentioned, along with other important characteristics: were messages selected from particular individuals, written by the individuals themselves, or did they also include messages that amplify or replicate news items or articles found online?”

**Answer 5:** Thank you for this valuable comment, which I fully agree with, as it helped to clarify an important aspect of the study design. In response, the methods section has been revised to specify that the analysed material consisted of original user-generated posts containing individuals’ own interpretations of numerical or statistical information, while posts that merely reproduced external content without interpretation were excluded.

**Comment 6:** “The recommendations included at the end of the article seem appropriate to me, but I think they lack concreteness: in what specific ways can they be implemented? I also miss the inclusion of some references illustrating how they have been previously tested or included in interventions. Additionally, given the exploratory nature of the analyses, I would add here a comment to remind the reader that the causal interpretation (in which the recommendations are based) is not granted.”

**Answer 6:** Thank you for this comment. In response, the recommendations section has been revised to provide greater concreteness by outlining how they can be incorporated into existing public health communication practices and institutional frameworks. In addition, selected references have been included to illustrate prior applications, and a clarification has been added to emphasise the exploratory nature of the analyses and the non-causal interpretation of the proposed recommendations.

**Comment 7:** “The enumeration of the ten errors in the introduction is somewhat difficult to follow. I suggest using a different, more visual format, such as a table or a figure, in which the ten errors are clearly presented, what they consist of, and (this is important) an example of how a message containing that type of error would be formulated. I believe this would greatly contribute to a better understanding of the research approach.”

**Answer 7:** Table 1 has been added, presenting the ten categories of statistical misreasoning together with their definitions and illustrative examples to provide a clearer and more accessible overview of the coding framework.

**Comment 8:** “I encourage the author to share the study data in an anonymized manner, as well as other materials that might be useful for replicating the study or motivating new research.”

**Answer 8:** Thank you for this helpful suggestion. In response, I have provided an anonymized Excel file in which each column corresponds to a category. A value of 0 indicates that the category was not assigned to a given post, while a value of 1 indicates that it was present. I have also updated the data availability statement accordingly.

**Response to Reviewer #2:**

**Dear Reviewer nr 2,**

**Comment 1:** “The authors set out to count various types of failures in statistical reasoning used to advocate against COVID vaccination in a sample of Facebook posts. They present which types of errors commonly occur and co-occur and proceed to lay out a set of recommendations to reduce these types of errors. Generally speaking I think this type of data should be published and available, there is value here despite the relative simplicity of what it can contribute theoretically and practically. However, I have some major issues with the current write-up which you will find below.”

**Answer 1**: Thank you for your thoughtful review and valuable comments, which have helped improve the clarity and quality of the manuscript.

**Comment 2:** “First, I find the methodological detail lacking here. The authors state that "posts were collected from online spaces discussing opposition to vaccination or criticizing immunization policies" - given that the results show that there were no posts which do not contain statistical mis-reasoning - was post selection aimed exclusively at posts which argue against vaccination? This should be clearly stated. Further, the selection criteria seems not to have much systematicity to it - how was this done. Were all posts within a time period scraped - then criteria applied to retain those which adhere? Was an author simply scrolling and selecting based on the criteria but doing so "on the fly"?”

**Answer 2:** In response to another reviewer’s comment, a detailed clarification of the coding procedure was added, including how categories were defined, applied systematically across the dataset, and developed through an iterative process to ensure conceptual completeness without redundancy. In addition, the description of data collection was clarified to explicitly state that the sample was restricted to posts expressing opposition to vaccination, as well as to emphasise the purposive nature of the sampling and the systematic application of predefined inclusion and exclusion criteria.

**Comment 3:** “Second, it is unclear whether posts come from unique members or whether some members of these discussion groups contributed multiple posts? If posts come from unique posters then fine - however if the posts are not independent then the current analysis and the one I propose becomes problematic. E.g., if a single member posts twice but does not repeat the same mis-reasoning errors then there is an argument to be made that these should be combined in a single "argument" rather than separate "posts" as for that particular person, for their thought stream and argumentation there is co-occurrence of errors even if they do not occur within the same post.”

**Answer 3:** Thank you for this important and insightful comment. The analysis in this study was conducted at the level of individual posts. A limitation has been added to acknowledge that multiple posts may have originated from the same individual, including anonymous users whose identities could not be consistently traced. This limitation and its potential implications for the independence of observations are now explicitly discussed in the manuscript.

**Comment 4:** “Third, and the author touches on this as a limitation - what was the inductive approach to coding? Was it an established qualitative technique which then led to quantification after coding or was it not an established technique and protocol? For example, the posts could have been analysed initially by coding each statistical mis-reasoning error into an initial code - a very micro level where almost every code would be independent of each other - then in a second pass the micro-codes can be categorized into more broad categories - and then perhaps after a couple of passes of this the data would reveal what categories are present - whether there is redundancy/overlap and then pruning could be applied to end up with the categories. On the other hand it could have been pretty much a priori definition of categories before coding. It is unclear what was exactly done.”

**Answer 4:** In response to another reviewer’s comment, the description of the coding procedure was expanded to clarify how categories were defined and applied consistently across the dataset, how they were developed through an iterative, inductive process of identifying and refining patterns of misreasoning, and to present the final set of categories with definitions and illustrative examples in Table 1. The description has also been further clarified to explicitly outline the stepwise coding process, including the initial identification of individual instances of misreasoning and their subsequent iterative grouping and refinement into broader categories.

**Comment 5:** “Fourth, the statistical analysis seems arbitrary. Why are counts compared to 50%? No rationale is given for why 50% is a reasonable comparator. A % could be defined as practically interesting - e.g., does an error occur more than 10% of the time (statistically significantly) or 0% or based on the number of posts what number (binomial distribution probability) would be required to state it occurs more than X% of the time. Though this may not be necessary at all. I would be more interested in formal statistical comparison of whether error A occurs more often than error B, C, etc. Considering there is also co-occurrence - Cochran Q as an omnibus test (though not even necessary really as there is clearly significant difference as function of type of error) and follow-up pairwise McNemar tests to look at differences between errors (adjusted for multiple comparisons). E.g., is the correlation-causation fallacy more prevalent than base rate neglect, statistically? The additional co-occurrence analysis through phi coefficient computation is both somewhat clear and a bit redundant in my view. And all of the reported phi values imply quite small co-occurrence - indeed I would call some practically not interesting. Also, it is not clear from the text whether the sole criterium for reporting was statistical significance as the text mentions "negligible" coefficients as well.”

**Answer 5:** Thank you for this thoughtful and helpful comment. The manuscript has been revised to clarify the rationale for the use of the 50% threshold, which is now explicitly described as an indicator of majority occurrence used to identify dominant patterns of misreasoning, rather than as a theoretically derived benchmark. In addition, the interpretation of phi coefficients has been clarified to emphasise that only associations that were either statistically significant or of non-negligible magnitude were retained, thereby accounting for both statistical and practical relevance. The statistical analysis has also been more clearly framed as primarily descriptive and exploratory, aimed at identifying dominant patterns and structural relationships rather than conducting exhaustive pairwise comparisons between categories.

**Comment 6:** “There are also some issues with the inferences being drawn. E.g., the author states “The findings of this study offer a new perspective, demonstrating that the persistence of inaccurate beliefs is driven to a significant extent by recurring patterns of errors in reasoning about numbers, risk, and statistical relationships.”. The data here does not allow for this conclusion – “persistence of inaccurate beliefs” was not measured – nor were other sources reinforcing inaccurate beliefs taken into account – the pattern could very may be rationalized by seeking out poor stats not driven by poor statistical reasoning (indeed, I expect this to be the case more often than not – people have beliefs, ideology, political views and seek out information that confirms their beliefs – rather than genuinely looking into data and their beliefs being constructed based on faulty statistical reasoning. The author states something similar in the methods section. The inferences should be limited to what the data shows - and the data simply counts the types of statistical reasoning failures when arguing against vaccination - not how much of that argumentation is driven by the errors or how persistent this is. It also doesn't tell us in general "how numerical data is processed" when discussing vaccines - as we have no comparator - we do not know what is the prevalence of these errors in general - just in one, extremely biased set of posts/groups.”

**Answer 6:** The manuscript has been revised to remove statements suggesting that statistical misreasoning drives the persistence or formation of inaccurate beliefs, both at the beginning of the discussion and in the conclusions.

**Comment 7:** “I like the recommendations set out by the author but most of the first part of the discussion simply restates, verbatim, the results - the reader has seen the table of occurrences and the histogram etc. - repeating all the results without added value of discussing the results deeper is simply redundant. I would be fine with not forcing a long discussion session - the results are very simple, there is not much theoretical that can be inferred or added - the highlight of the discussion are the recommendations - so that (and limitations) are what can be left in there with only a very short recap of results.”

**Answer 7:** The discussion section has been revised and repetitive descriptions of the results have been removed, while retaining only a brief summary of the key findings and focusing more on their interpretation.

**Comment 8:** “I would also like to see explicit examples of all 10 errors, from actual posts analysed. The author gives one example in which three errors co-occur - but seeing as this paper would ultimately be aimed at people unfamiliar with cognitive biases and reasoning errors more than to others - explicit examples would be very welcome. I also strongly recommend making data available. The author states there is no identifiable information and therefore no ethics approval was necessary but then that some restrictions would apply and therefore no data was shared. At the very least, the counts for each error in each post can be shared - the analytical dataset. But in reality the posts themselves can easily be further anonymised - removing dates, times, group information, usernames, mention of locations and such form the posts themselves. I expect one of these options (and perhaps the SPSS syntax used to analyse the data) to be made available (data, whatever the level of it, should be in a non-proprietary format, .csv preferably).”

**Answer 8:** In response to another reviewer’s comments, Table 1 was added to present all ten categories together with their definitions and illustrative examples, and an anonymized analytical dataset has been provided, with the data availability statement updated accordingly. Due to ethical considerations and the potential risk of indirect identification, the original posts cannot be shared, even in anonymized form.

**Comment 9:** “A nod to the vast cognitive biases, reasoning and decision making literature could be made in the intro/discussion as well - and could serve to flesh out the discussion in terms of how results fit into lab-based research and theory if need be (there are a couple of references but very little).”

**Answer 9:** The discussion section has been revised to include additional references to the broader literature on cognitive biases and decision-making, thereby better situating the findings within established theoretical and empirical research.
