# Supplementary material for: Statistical misreasoning in online content about vaccines: Implications and recommendations for addressing disinformation
Source: PLoS One. 2026 Aug 3;21(8):e0355341. doi: 10.1371/journal.pone.0355341 (PMC13432100; doi:10.1371/journal.pone.0355341)
Supplement: Attachment — Submitted filename: Point-by-point-response MAY.2026.docx [file pone.0355341.s004.docx]

**Point-by-point response template**

**Date: 22.05.2026**

**Manuscript Number:** PONE-D-25-65889R1

**Title of Article:** “Statistical misreasoning in online content about vaccines: implications and recommendations for addressing disinformation”

**Name of the Corresponding Author: Michal Ordak**

**Email Address of the Corresponding Author:** [**michal.ordak@wum.edu.pl**](mailto:michal.ordak@wum.edu.pl)

Dear Prof. Emily Chenette,

Thank you for sending the Reviewer’s comments regarding my paper entitled “Statistical misreasoning in online content about vaccines: implications and recommendations for addressing disinformation”, by M. Ordak (PONE-D-25-65889R1). I greatly appreciate all valuable suggestions proposed by the Reviewer, which I consider while preparing the revised version of the manuscript.

**Specific Responses**

**Response to Reviewer #1:**

**Dear Reviewer nr 1,**

**Comment 1:** “I have read the revision of this manuscript. I think all my comments have been addressed. The study has several problems and limitations that affect the generalization of the conclusions. But now that the limitations of the study are clearer, the reader will be able to judge the relevance of the results. If anything, the 50% threshold for the frequency analysis is still not well justified, so I would ask to include a brief explanation either in the Results or in the Discussion sections.”

**Answer 1:** Thank you for your positive evaluation of the revised manuscript and for acknowledging that the previous comments have been adequately addressed. In response to your remaining suggestion, I added a brief clarification in the Results section explaining that the 50% threshold was treated as a pragmatic indicator of majority occurrence within the analysed sample and was used to distinguish dominant from less prevalent forms of statistical misreasoning.

**Response to Reviewer #2:**

**Dear Reviewer nr 2,**

**Comment 1:** “The detailed and comprehensive responses from the author are much appreciated. However, in light of comments from my fellow reviewer and the responses themselves, I have to say I still some issues with the current write-up. I am assigning this a "major revision" recommendation. I see the overall message as valuable, but at the same time what is here in methodological and other terms is quite limited and with the added core issues regarding the workflow - even if I would like more awareness of these biases through more publications - I cannot recommend publishing in this form.”

**Answer 1**: Thank you for your detailed evaluation of the manuscript and for acknowledging the value and relevance of the overall message of the study. I also appreciate your additional comments and methodological suggestions, which once again contributed to improving the clarity and quality of the manuscript. All remarks were carefully considered and addressed in the revised version.

**Comment 2:** “Table 1 adds to the clarity but also clearly shows potential issues of categories not being well delineated.

For example. The "Cherry picking" descriptor states "selective use of numerical evidence (e.g., specific datasets, countries or time periods)...". But the example of denominator neglect includes reference to a specific time period so that example is a compound. Sure the emphasis is on "200 cases of complications" without taking into account the overall number of vaccinated individual but it is also aimed at a specific time period ("last month"). Likewise, small sample fallacy could be seen as cherry picking ("a few people I know" being cherry picked and the vast other information available being ignored). Perhaps I'm getting caught on the naming here - if it were "anecdotal personal experience" rather than "small sample fallacy" for this example then it would be better. One could argue that the example in cherry picking is also a compound of multiple biases. Yes the emphasis is on "one selected country" - but really the rest of it includes other biases. Linking the number of cases and number of vaccinations in a causal relationship for one, ignoring that testing is more frequent and in full swing at that point, that measures were relaxed, ignoring the make up of those new cases etc. Now, it may be the case that these were indeed counted as co-occurrences as only 8% of the posts had a single bias - but it's unclear whether this is the case or if coding issues produced these overlaps. There are other examples between the various categories.”

**Answer 2:** Thank you for this helpful observation. To clarify the intended relationship between categories, the manuscript was revised to explicitly state that the coding categories were not treated as fully mutually exclusive, as individual posts frequently contained overlapping forms of statistical misreasoning. In addition, several formulations emphasizing “non-redundancy” and avoidance of overlap were replaced with wording focused on “analytical distinctiveness”, “analytically differentiated typology” and minimizing “conceptual redundancy” in order to better reflect the analytical purpose of the framework and the compound nature of many examples presented in Table 1.

**Comment 3:** “I would also echo Reviewer 1 that it would make sense if additional raters were invited at this stage with the categories identified and given the posts to categorize and produce a measure of inter-rater reliability. Even with the edits, I do not find the explanation of the process very satisfying. Although it is more transparent - it does not increase my confidence in the reliability of the work.”

**Answer 3:** Thank you for this important observation. In response, the limitations section was expanded to more explicitly acknowledge that the absence of independent coding prevents formal assessment of the reproducibility and reliability of classification decisions. The revised manuscript now clarifies that the proposed framework should be regarded as exploratory and theory-generating rather than as a fully validated and independently reproducible coding system, and it explicitly identifies independent coding procedures and formal inter-rater reliability assessment as important directions for future validation of the framework.

**Comment 4:** “There are still places in the manuscript where clarity can be better. The phrase “statistically significant or of non-negligible magnitude were retained” but from what is written in text it seems the criterion was a p < 0.05 so the "or of non-negligible magnitude" should be removed, or if it was somehow taken into account - then a threshold should be introduced rather than the term "non-negligible.”

**Answer 4:** Thank you for this comment. In response, I revised the relevant sentence in the Results section by removing the reference to “non-negligible magnitude” so that the interpretation criteria are fully consistent with the statistical significance threshold (p < 0.05) described in the manuscript.

**Comment 5:** “Now that the 50% is explained as being the comparator for prevalence frequencies - it seems like a lot of fluff to simply state biases "X and Y occur in the majority of posts". The chi-squared tests seem like a tack-on which do not add much to the results or interpretations.”

**Answer 5:** Thank you for this valuable comment. In response, I revised the wording in the Results section to reduce the emphasis placed on the chi-square tests and to better reflect the primarily descriptive nature of the analysis. In addition, following the suggestion of the first reviewer, I added a clarifying sentence explaining that the 50% threshold was treated as a pragmatic indicator of majority occurrence within the analysed sample and was used to distinguish dominant from less prevalent forms of statistical misreasoning.

**Comment 6:** “The author states in the discussion that co-occurrence may indicate "structural links" - however, it may simply be high salience - some biases are strong and prevalent, very salient - so these may be more prevalent and co-occur for that reason, not necessarily because there is a structural link (though there is a more theoretical discussion to be had on whether all biases are really at their root the same cognitive process etc.).”

**Answer 6:** I revised the discussion section to avoid overly strong interpretations of co-occurrence patterns as evidence of structural links between biases and clarified that the observed co-occurrences may also reflect the high prevalence and salience of particular forms of statistical misreasoning.

**Comment 7:** “Between 2023 and November 2025” - at least the month in 2023 should be in there to match how 2025 is reported - though if posts are not to be shared openly then there is no reason not to have exact dates in there.”

**Answer 7:** I clarified the timeframe of data collection by specifying both the starting and ending months of the analysed period.

**Comment 8:** “There are two Table 1-s – the new table was added without adjusting numeration of the other tables.”

**Answer 8:** Thank you for this valid comment. In response, the table numbering has been corrected.

**Comment 9:** “I am still unconvinced by the data sharing statements. I would suggest pointing to exact TOS sections and perhaps having the "available upon reasonable request" type of statement in there if the data cannot be shared openly.”

**Answer 9:** I revised the data availability statement to clarify that the original Facebook posts are not publicly shared due to privacy considerations and restrictions related to Facebook Terms of Service governing the redistribution of user-generated content.
