# Supplementary material for: Statistical misreasoning in online content about vaccines: Implications and recommendations for addressing disinformation
Source: PLoS One. 2026 Aug 3;21(8):e0355341. doi: 10.1371/journal.pone.0355341 (PMC13432100; doi:10.1371/journal.pone.0355341)
Supplement: Attachment — Submitted filename: Point-by-point-response July 2026.docx [file pone.0355341.s005.docx]

**Point-by-point response template**

**Date: 18.07.2026**

**Manuscript Number:** PONE-D-25-65889R2

**Title of Article:** “Statistical misreasoning in online content about vaccines: implications and recommendations for addressing disinformation”

**Name of the Corresponding Author: Michal Ordak**

**Email Address of the Corresponding Author:** [**michal.ordak@wum.edu.pl**](mailto:michal.ordak@wum.edu.pl)

Dear Prof. Emily Chenette,

Thank you for sending the Reviewer’s comments regarding my paper entitled “Statistical misreasoning in online content about vaccines: implications and recommendations for addressing disinformation”, by M. Ordak (PONE-D-25-65889R2). I greatly appreciate all valuable suggestions proposed by the Reviewer, which I consider while preparing the revised version of the manuscript.

**Specific Responses**

**Response to Reviewer #1:**

**Dear Reviewer nr 1,**

**Comment 1:** “I have read the revised version of the manuscript.

All of my previous comments have been adequately addressed. The only remaining issue was the justification for the 50% threshold, which is now included. While I do not think the "pragmatic" rationale is entirely convincing, I believe it is acceptable as long as it is clearly and transparently reported. Overall, although the study has several limitations (some of which were appropriately highlighted by the other reviewer), I think these limitations are adequately acknowledged and discussed, so readers are unlikely to be misled.

Minor comment: I noticed some inconsistencies in the use of decimal notation (e.g., 0.001 vs. 0,001). Please ensure that the notation is consistent throughout the manuscript.”

**Answer 1:** I thank the Reviewer for the positive assessment and for confirming that my revisions have adequately addressed the previous comments. I appreciate the Reviewer's thoughtful evaluation and positive overall assessment of the manuscript. Thank you for pointing this out. I have corrected the decimal notation in the p values throughout the manuscript to ensure consistency.

**Response to Reviewer #2:**

**Dear Reviewer nr 2,**

**Comment 1:** “I appreciate the author's responses. I still feel that the chi-squared tests versus a 50% thresholds are not all that informative but they also don't really hurt the paper (except for making it longer). A minor formatting issue is that some p values in the table have decimal commas, rather than dots so just general polish of these types of things to make it cleaner is probably necessary. Considering that the main methodological issues cannot be solved without additional workload and that the author addresses this as an issue in the discussion, I am leaving it to the editor to decide whether Table 1 and the methodological weaknesses require more work or if addressing these points as they have been is enough. Therefore I'm recommending "minor revisions" but if the editor decides that additional work is not required I do not expect to get this back on my proverbial desk and in that case consider the recommendation as "accept".”

**Answer 1**: Thank you for your positive evaluation of the manuscript and for your constructive comments. Thank you for acknowledging that the main methodological limitations have been addressed in the Discussion. In accordance with your comment, I have revised the formatting of the p values throughout the manuscript by replacing decimal commas with decimal points to ensure consistent notation.
