# Supplementary material for: Statistical misreasoning in online content about vaccines: Implications and recommendations for addressing disinformation
Source: PLoS One. 2026 Aug 3;21(8):e0355341. doi: 10.1371/journal.pone.0355341 (PMC13432100; doi:10.1371/journal.pone.0355341)
Supplement: Peer review history

# Peer review: Decision Letter 0

**DOI:** 10.1371/journal.pone.0355341.r001

**Authors:** José C. Perales

6 Mar 2026

PONE-D-25-65889

Statistical misreasoning in online content about vaccines: implications and recommendations for addressing disinformation

PLOS One

Dear Dr. Ordak,

Thank you for submitting your manuscript to PLOS ONE. After careful consideration, we find that it has merit but does not fully meet our publication criteria in its current form. We therefore invite you to submit a revised version that addresses the points raised during the review process.

The reviewers have identified three major concerns.

First, it is unclear how the labelling system used to define the ten statistical errors was developed. Tools of this kind customarily undergo a process of gradual refinement to minimise the risk of distorting the observed reality, or of the number of labels being arbitrary and thus susceptible to selection bias. Such refinement also guards against overlooking conceptual overlap between labels or, conversely, failing to distinguish between instances assigned to the same label.

Second, no measures are described to ensure the reliability of the coding system. Qualitative analyses customarily include safeguards against subjectivity in coding. As noted by one reviewer, this requires, at minimum, that several independent coders apply the same instrument to the same pool of instances, with convergence formally assessed and documented.

Third, both reviewers flag the presence of overstatements. While they recognise the value of the descriptive analyses provided, they note that certain assertions are not adequately grounded in the evidence presented.

Please note that addressing these principal concerns, together with the additional points raised by the reviewers, will require substantial revision, and the prospects for publication remain uncertain. If you are prepared to undertake such revisions, please submit your revised manuscript by Apr 20 2026 11:59PM. If you will need more time than this to complete your revisions, please reply to this message or contact the journal office at plosone@plos.org. When you're ready to submit your revision, log on to https://www.editorialmanager.com/pone/ and select the 'Submissions Needing Revision' folder to locate your manuscript file.

Please include the following items when submitting your revised manuscript:

- A letter that responds to each point raised by the academic editor and reviewer(s). You should upload this letter as a separate file labeled 'Response to Reviewers'.
- A marked-up copy of your manuscript that highlights changes made to the original version. You should upload this as a separate file labeled 'Revised Manuscript with Track Changes'.
- An unmarked version of your revised paper without tracked changes. You should upload this as a separate file labeled 'Manuscript'.

If you would like to make changes to your financial disclosure, please include your updated statement in your cover letter. Guidelines for resubmitting your figure files are available below the reviewer comments at the end of this letter.

If applicable, we recommend that you deposit your laboratory protocols in protocols.io to enhance the reproducibility of your results. Protocols.io assigns your protocol its own identifier (DOI) so that it can be cited independently in the future. For instructions see: https://journals.plos.org/plosone/s/submission-guidelines#loc-laboratory-protocols. Additionally, PLOS ONE offers an option for publishing peer-reviewed Lab Protocol articles, which describe protocols hosted on protocols.io. Read more information on sharing protocols at https://plos.org/protocols?utm_medium=editorial-email&utm_source=authorletters&utm_campaign=protocols.

We look forward to receiving your revised manuscript.

Kind regards,

José C. Perales

Academic Editor

PLOS One

Journal Requirements:

When submitting your revision, we need you to address these additional requirements.

1.Please ensure that your manuscript meets PLOS ONE's style requirements, including those for file naming. The PLOS ONE style templates can be found at

https://journals.plos.org/plosone/s/file?id=wjVg/PLOSOne_formatting_sample_main_body.pdf and

https://journals.plos.org/plosone/s/file?id=ba62/PLOSOne_formatting_sample_title_authors_affiliations.pdf

2. In your Methods section, please include additional information about your dataset and ensure that you have included a statement specifying whether the collection and analysis method complied with the terms and conditions for the source of the data.

3. We note that you have indicated that there are restrictions to data sharing for this study. PLOS only allows data to be available upon request if there are legal or ethical restrictions on sharing data publicly. For more information on unacceptable data access restrictions, please see http://journals.plos.org/plosone/s/data-availability#loc-unacceptable-data-access-restrictions.

Before we proceed with your manuscript, please address the following prompts:

a) If there are ethical or legal restrictions on sharing a de-identified data set, please explain them in detail (e.g., data contain potentially identifying or sensitive patient information, data are owned by a third-party organization, etc.) and who has imposed them (e.g., a Research Ethics Committee or Institutional Review Board, etc.). Please also provide contact information for a data access committee, ethics committee, or other institutional body to which data requests may be sent.

b) If there are no restrictions, please upload the minimal anonymized data set necessary to replicate your study findings to a stable, public repository and provide us with the relevant URLs, DOIs, or accession numbers. For a list of recommended repositories, please see

https://journals.plos.org/plosone/s/recommended-repositories. You also have the option of uploading the data as Supporting Information files, but we would recommend depositing data directly to a data repository if possible.

We will update your Data Availability statement on your behalf to reflect the information you provide.

4. If the reviewer comments include a recommendation to cite specific previously published works, please review and evaluate these publications to determine whether they are relevant and should be cited. There is no requirement to cite these works unless the editor has indicated otherwise.

[Note: HTML markup is below. Please do not edit.]

Reviewers' comments:

Reviewer's Responses to Questions

**Comments to the Author**

1. Is the manuscript technically sound, and do the data support the conclusions?

The manuscript must describe a technically sound piece of scientific research with data that supports the conclusions. Experiments must have been conducted rigorously, with appropriate controls, replication, and sample sizes. The conclusions must be drawn appropriately based on the data presented.

Reviewer #1: Partly

Reviewer #2: Partly

**********

2. Has the statistical analysis been performed appropriately and rigorously?

Reviewer #1: Yes

Reviewer #2: No

**********

3. Have the authors made all data underlying the findings in their manuscript fully available?

The PLOS Data policy requires authors to make all data underlying the findings described in their manuscript fully available without restriction, with rare exception (please refer to the Data Availability Statement in the manuscript PDF file). The data should be provided as part of the manuscript or its supporting information, or deposited to a public repository. For example, in addition to summary statistics, the data points behind means, medians and variance measures should be available. If there are restrictions on publicly sharing data—e.g. participant privacy or use of data from a third party—those must be specified.

Reviewer #1: No

Reviewer #2: No

**********

4. Is the manuscript presented in an intelligible fashion and written in standard English?

PLOS ONE does not copyedit accepted manuscripts, so the language in submitted articles must be clear, correct, and unambiguous. Any typographical or grammatical errors should be corrected at revision, so please note any specific errors here.

Reviewer #1: Yes

Reviewer #2: Yes

**********

5. Review Comments to the Author

Please use the space provided to explain your answers to the questions above. You may also include additional comments for the author, including concerns about dual publication, research ethics, or publication ethics. (Please upload your review as an attachment if it exceeds 20,000 characters)

Reviewer #1: This manuscript describes a study in which hundreds of messages about vaccines posted on Facebook are examined, with the aim of studying the patterns in the statistical errors that underpin many of these messages.

The study addresses a topic of high interest and tackles a complex problem. I also consider the approach based on statistical errors or misreasoning (rather than on prior beliefs or ideological issues) to be original and valuable. Additionally, I appreciate that the article is very direct and straightforward.

On the other hand, there are some methodological limitations that affect the conclusions of the study, meaning that, in any case, it should be interpreted merely for descriptive purposes. Below I explain the main limitations I found in my reading and comment on some possible improvements.

--

1. The main problem of this study is that, at least with the information included in the article, it does not seem sufficiently protected against confirmation bias on the part of the researcher. The content and style of the messages are supposed to reveal ten different statistical errors. It is stated that the ten categories of statistical errors were defined a priori. But were the specific elements or the type of language that reveals each of these errors also defined a priori? If not, there is a risk that the data will be interpreted (that is, messages classified as belonging to one category or another) according to the reader’s expectations. In this regard, both the a priori definition of key message characteristics and the use of a classification rubric, as well as the collaboration of several judges, would have been necessary measures to ensure an adequate interpretation of the data. If these measures were not taken in advance, perhaps the dataset can be now examined by a new team of judges by using a pre-defined set of rules or rubric, so that the conclusions are more robust?

2. Similarly: it should also be explained in more detail how the categories were chosen. The justification in the article refers to their number (fewer than 10 would imply that the list is not comprehensive, more than 10 would lead to overlap between categories), but ten is still an arbitrary number (why not 9? 11?) and there is no reasoned explanation or theoretical justification for this decision. Surely the author can provide more information in this respect so that the explanation in the revised article is clear.

3. The analysis of structural patterns of misreasoning seems potentially useful at a descriptive level, but it has limitations. For example, it is stated (or inferred from the reading of the article) that this analysis may reveal something useful about the structure of misinformation narratives and discourses, or even about human cognition, but in any case this is indirect evidence that can be interpreted in several ways. To begin with, as far as I understand, it is an exploratory analysis for which there is no clear theory-based hypothesis. That is, it is not proposed a priori that there are certain types of errors that have some kind of association and are therefore expected to appear together frequently. If such hypotheses existed, they should be made explicit in the article.

4. The analysis of the co-occurrence of different errors within the same message has other limitations, such as not controlling for message length. Since this study is based on Facebook data and not a microblogging network with fixed character limits (e.g., Twitter), I imagine that message length, measured in number of words, will be highly variable. My reasoning is that the longer the message, the greater the probability of combining several errors in a single message, which could better contextualize the results of this analysis, but this possibility has not been explored.

5. In general, I believe that important information is missing to understand how the research was conducted. It has already been mentioned that there are no references to any system or rubric for classifying messages. Nor is message length mentioned, along with other important characteristics: were messages selected from particular individuals, written by the individuals themselves, or did they also include messages that amplify or replicate news items or articles found online?

6. The recommendations included at the end of the article seem appropriate to me, but I think they lack concreteness: in what specific ways can they be implemented? I also miss the inclusion of some references illustrating how they have been previously tested or included in interventions. Additionally, given the exploratory nature of the analyses, I would add here a comment to remind the reader that the causal interpretation (in which the recommendations are based) is not granted.

7. The enumeration of the ten errors in the introduction is somewhat difficult to follow. I suggest using a different, more visual format, such as a table or a figure, in which the ten errors are clearly presented, what they consist of, and (this is important) an example of how a message containing that type of error would be formulated. I believe this would greatly contribute to a better understanding of the research approach.

8. I encourage the author to share the study data in an anonymized manner, as well as other materials that might be useful for replicating the study or motivating new research.

Reviewer #2: The authors set out to count various types of failures in statistical reasoning used to advocate against COVID vaccination in a sample of Facebook posts. They present which types of errors commonly occur and co-occur and proceed to lay out a set of recommendations to reduce these types of errors.

Generally speaking I think this type of data should be published and available, there is value here despite the relative simplicity of what it can contribute theoretically and practically. However, I have some major issues with the current write-up which you will find below.

First, I find the methodological detail lacking here. The authors state that "posts were collected from online spaces discussing opposition to vaccination or criticizing immunization policies" - given that the results show that there were no posts which do not contain statistical mis-reasoning - was post selection aimed exclusively at posts which argue against vaccination? This should be clearly stated. Further, the selection criteria seems not to have much systematicity to it - how was this done. Were all posts within a time period scraped - then criteria applied to retain those which adhere? Was an author simply scrolling and selecting based on the criteria but doing so "on the fly"?

Second, it is unclear whether posts come from unique members or whether some members of these discussion groups contributed multiple posts? If posts come from unique posters then fine - however if the posts are not independent then the current analysis and the one I propose becomes problematic. E.g., if a single member posts twice but does not repeat the same mis-reasoning errors then there is an argument to be made that these should be combined in a single "argument" rather than separate "posts" as for that particular person, for their thought stream and argumentation there is co-occurrence of errors even if they do not occur within the same post.

Third, and the author touches on this as a limitation - what was the inductive approach to coding? Was it an established qualitative technique which then led to quantification after coding or was it not an established technique and protocol? For example, the posts could have been analysed initially by coding each statistical mis-reasoning error into an initial code - a very micro level where almost every code would be independent of each other - then in a second pass the micro-codes can be categorized into more broad categories - and then perhaps after a couple of passes of this the data would reveal what categories are present - whether there is redundancy/overlap and then pruning could be applied to end up with the categories. On the other hand it could have been pretty much a priori definition of categories before coding. It is unclear what was exactly done.

Fourth, the statistical analysis seems arbitrary. Why are counts compared to 50%? No rationale is given for why 50% is a reasonable comparator. A % could be defined as practically interesting - e.g., does an error occur more than 10% of the time (statistically significantly) or 0% or based on the number of posts what number (binomial distribution probability) would be required to state it occurs more than X% of the time. Though this may not be necessary at all. I would be more interested in formal statistical comparison of whether error A occurs more often than error B, C, etc. Considering there is also co-occurrence - Cochran Q as an omnibus test (though not even necessary really as there is clearly significant difference as function of type of error) and follow-up pairwise McNemar tests to look at differences between errors (adjusted for multiple comparisons). E.g., is the correlation-causation fallacy more prevalent than base rate neglect, statistically? The additional co-occurrence analysis through phi coefficient computation is both somewhat clear and a bit redundant in my view. And all of the reported phi values imply quite small co-occurrence - indeed I would call some practically not interesting. Also, it is not clear from the text whether the sole criterium for reporting was statistical significance as the text mentions "negligible" coefficients as well.

There are also some issues with the inferences being drawn. E.g., the author states “The findings of this study offer a new perspective, demonstrating that the persistence of inaccurate beliefs is driven to a significant extent by recurring patterns of errors in reasoning about numbers, risk, and statistical relationships.”. The data here does not allow for this conclusion – “persistence of inaccurate beliefs” was not measured – nor were other sources reinforcing inaccurate beliefs taken into account – the pattern could very may be rationalized by seeking out poor stats not driven by poor statistical reasoning (indeed, I expect this to be the case more often than not – people have beliefs, ideology, political views and seek out information that confirms their beliefs – rather than genuinely looking into data and their beliefs being constructed based on faulty statistical reasoning. The author states something similar in the methods section. The inferences should be limited to what the data shows - and the data simply counts the types of statistical reasoning failures when arguing against vaccination - not how much of that argumentation is driven by the errors or how persistent this is. It also doesn't tell us in general "how numerical data is processed" when discussing vaccines - as we have no comparator - we do not know what is the prevalence of these errors in general - just in one, extremely biased set of posts/groups.

I like the recommendations set out by the author but most of the first part of the discussion simply restates, verbatim, the results - the reader has seen the table of occurrences and the histogram etc. - repeating all the results without added value of discussing the results deeper is simply redundant. I would be fine with not forcing a long discussion session - the results are very simple, there is not much theoretical that can be inferred or added - the highlight of the discussion are the recommendations - so that (and limitations) are what can be left in there with only a very short recap of results.

I would also like to see explicit examples of all 10 errors, from actual posts analysed. The author gives one example in which three errors co-occur - but seeing as this paper would ultimately be aimed at people unfamiliar with cognitive biases and reasoning errors more than to others - explicit examples would be very welcome. I also strongly recommend making data available. The author states there is no identifiable information and therefore no ethics approval was necessary but then that some restrictions would apply and therefore no data was shared. At the very least, the counts for each error in each post can be shared - the analytical dataset. But in reality the posts themselves can easily be further anonymised - removing dates, times, group information, usernames, mention of locations and such form the posts themselves. I expect one of these options (and perhaps the SPSS syntax used to analyse the data) to be made available (data, whatever the level of it, should be in a non-proprietary format, .csv preferably).

A nod to the vast cognitive biases, reasoning and decision making literature could be made in the intro/discussion as well - and could serve to flesh out the discussion in terms of how results fit into lab-based research and theory if need be (there are a couple of references but very little).

**********

6. PLOS authors have the option to publish the peer review history of their article (what does this mean?). If published, this will include your full peer review and any attached files.

If you choose “no”, your identity will remain anonymous but your review may still be made public.

**Do you want your identity to be public for this peer review?**  For information about this choice, including consent withdrawal, please see our Privacy Policy.

Reviewer #1: No

Reviewer #2: No

**********

[NOTE: If reviewer comments were submitted as an attachment file, they will be attached to this email and accessible via the submission site. Please log into your account, locate the manuscript record, and check for the action link "View Attachments". If this link does not appear, there are no attachment files.]

To ensure your figures meet our technical requirements, please review our figure guidelines: https://journals.plos.org/plosone/s/figures

You may also use PLOS’s free figure tool, NAAS, to help you prepare publication quality figures: https://journals.plos.org/plosone/s/figures#loc-tools-for-figure-preparation.

NAAS will assess whether your figures meet our technical requirements by comparing each figure against our figure specifications.

# Author comment: Author response to Decision Letter 1

**DOI:** 10.1371/journal.pone.0355341.r002

27 Mar 2026

Point-by-point response template

Date: 27.03.2026

Manuscript Number: PONE-D-25-65889

Title of Article: “Statistical misreasoning in online content about vaccines: implications and recommendations for addressing disinformation”

Name of the Corresponding Author: Michal Ordak

Email Address of the Corresponding Author: michal.ordak@wum.edu.pl

Dear Prof. Emily Chenette,

Thank you for sending the Reviewer’s comments regarding my paper entitled “Statistical misreasoning in online content about vaccines: implications and recommendations for addressing disinformation”, by M. Ordak (PONE-D-25-65889). I greatly appreciate all valuable suggestions proposed by the Reviewer, which I consider while preparing the revised version of the manuscript.

Specific Responses

Response to Academic Editor:

Comment 1: “The reviewers have identified three major concerns. First, it is unclear how the labelling system used to define the ten statistical errors was developed. Tools of this kind customarily undergo a process of gradual refinement to minimise the risk of distorting the observed reality, or of the number of labels being arbitrary and thus susceptible to selection bias. Such refinement also guards against overlooking conceptual overlap between labels or, conversely, failing to distinguish between instances assigned to the same label. Second, no measures are described to ensure the reliability of the coding system. Qualitative analyses customarily include safeguards against subjectivity in coding. As noted by one reviewer, this requires, at minimum, that several independent coders apply the same instrument to the same pool of instances, with convergence formally assessed and documented. Third, both reviewers flag the presence of overstatements. While they recognise the value of the descriptive analyses provided, they note that certain assertions are not adequately grounded in the evidence presented.”

Answer 1: Thank you for your synthesis of the reviewers’ comments and for identifying the three main areas of concern. I would like to indicate that these points have been addressed in the revised manuscript in line with the reviewers’ suggestions. The methods section has been revised to clarify how the categories of statistical misreasoning were developed and applied. The manuscript now also acknowledges that the coding was conducted by a single researcher and discusses this as a limitation, including the potential for subjectivity and confirmation bias. In addition, statements suggesting that statistical misreasoning drives the persistence or formation of inaccurate beliefs have been removed, and the exploratory nature of the analysis has been more clearly emphasised. I hope that these revisions address the concerns raised.

Comment 2: “In your Methods section, please include additional information about your dataset and ensure that you have included a statement specifying whether the collection and analysis method complied with the terms and conditions for the source of the data.”

Answer 2: The manuscript was revised to include a description of the data collection process and a statement confirming that the collection and analysis complied with the platform’s terms and conditions.

Response to Reviewer #1:

Dear Reviewer nr 1,

Comment 1: “The main problem of this study is that, at least with the information included in the article, it does not seem sufficiently protected against confirmation bias on the part of the researcher. The content and style of the messages are supposed to reveal ten different statistical errors. It is stated that the ten categories of statistical errors were defined a priori. But were the specific elements or the type of language that reveals each of these errors also defined a priori? If not, there is a risk that the data will be interpreted (that is, messages classified as belonging to one category or another) according to the reader’s expectations. In this regard, both the a priori definition of key message characteristics and the use of a classification rubric, as well as the collaboration of several judges, would have been necessary measures to ensure an adequate interpretation of the data. If these measures were not taken in advance, perhaps the dataset can be now examined by a new team of judges by using a pre-defined set of rules or rubric, so that the conclusions are more robust?”

Answer 1: Thank you for this insightful and important comment. The concern regarding potential confirmation bias and the transparency of coding decisions is well taken. In response, the methods section has been revised to clarify that coding decisions were based on identifying patterns of reasoning in the posts that corresponded to the conceptual definitions of each category, and that these criteria were applied consistently across the dataset. In addition, the Limitations section has been expanded to explicitly acknowledge that the coding was conducted by a single researcher without independent verification, which may introduce interpretive bias, including confirmation bias, and may affect the reproducibility of classification decisions. I hope that these clarifications address the reviewer’s concerns.

Comment 2: “Similarly: it should also be explained in more detail how the categories were chosen. The justification in the article refers to their number (fewer than 10 would imply that the list is not comprehensive, more than 10 would lead to overlap between categories), but ten is still an arbitrary number (why not 9? 11?) and there is no reasoned explanation or theoretical justification for this decision. Surely the author can provide more information in this respect so that the explanation in the revised article is clear.”

Answer 2: Thank you for this helpful comment. The point regarding the potential arbitrariness of the number of categories is well taken. In response, the methods section has been revised to clarify that the final number of categories was not predetermined but emerged from an iterative process of refining and grouping observed patterns of statistical misreasoning in the dataset. The revised text now explains that fewer categories led to a loss of important conceptual distinctions between different types of errors, whereas introducing additional categories resulted in conceptual overlap without adding analytical value. The set of ten categories therefore reflects a balance between conceptual completeness and non-redundancy.

Comment 3: “The analysis of structural patterns of misreasoning seems potentially useful at a descriptive level, but it has limitations. For example, it is stated (or inferred from the reading of the article) that this analysis may reveal something useful about the structure of misinformation narratives and discourses, or even about human cognition, but in any case this is indirect evidence that can be interpreted in several ways. To begin with, as far as I understand, it is an exploratory analysis for which there is no clear theory-based hypothesis. That is, it is not proposed a priori that there are certain types of errors that have some kind of association and are therefore expected to appear together frequently. If such hypotheses existed, they should be made explicit in the article.”

Answer 3: The methods section has been revised to clarify that the co-occurrence analysis is exploratory in nature and not based on pre-specified theory-driven hypotheses.

Comment 4: “The analysis of the co-occurrence of different errors within the same message has other limitations, such as not controlling for message length. Since this study is based on Facebook data and not a microblogging network with fixed character limits (e.g., Twitter), I imagine that message length, measured in number of words, will be highly variable. My reasoning is that the longer the message, the greater the probability of combining several errors in a single message, which could better contextualize the results of this analysis, but this possibility has not been explored.”

Answer 4: Thank you for this important observation. The limitations section has been expanded to acknowledge that message length was not controlled for and may influence the number and co-occurrence of errors identified within individual posts.

Comment 5: “In general, I believe that important information is missing to understand how the research was conducted. It has already been mentioned that there are no references to any system or rubric for classifying messages. Nor is message length mentioned, along with other important characteristics: were messages selected from particular individuals, written by the individuals themselves, or did they also include messages that amplify or replicate news items or articles found online?”

Answer 5: Thank you for this valuable comment, which I fully agree with, as it helped to clarify an important aspect of the study design. In response, the methods section has been revised to specify that the analysed material consisted of original user-generated posts containing individuals’ own interpretations of numerical or statistical information, while posts that merely reproduced external content without interpretation were excluded.

Comment 6: “The recommendations included at the end of the article seem appropriate to me, but I think they lack concreteness: in what specific ways can they be implemented? I also miss the inclusion of some references illustrating how they have been previously tested or included in interventions. Additionally, given the exploratory nature of the analyses, I would add here a comment to remind the reader that the causal interpretation (in which the recommendations are based) is not granted.”

Answer 6: Thank you for this comment. In response, the recommendations section has been revised to provide greater concreteness by outlining how they can be incorporated into existing public health communication practices and institutional frameworks. In addition, selected references have been included to illustrate prior applications, and a clarification has been added to emphasise the exploratory nature of the analyses and the non-causal interpretation of the proposed recommendations.

Comment 7: “The enumeration of the ten errors in the introduction is somewhat difficult to follow. I suggest using a different, more visual format, such as a table or a figure, in which the ten errors are clearly presented, what they consist of, and (this is important) an example of how a message containing that type of error would be formulated. I believe this would greatly contribute to a better understanding of the research approach.”

Answer 7: Table 1 has been added, presenting the ten categories of statistical misreasoning together with their definitions and illustrative examples to provide a clearer and more accessible overview of the coding framework.

Comment 8: “I encourage the author to share the study data in an anonymized manner, as well as other materials that might be useful for replicating the study or motivating new research.”

Answer 8: Thank you for this helpful suggestion. In response, I have provided an anonymized Excel file in which each column corresponds to a category. A value of 0 indicates that the category was not assigned to a given post, while a value of 1 indicates that it was present. I have also updated the data availability statement accordingly.

Response to Reviewer #2:

Dear Reviewer nr 2,

Comment 1: “The authors set out to count various types of failures in statistical reasoning used to advocate against COVID vaccination in a sample of Facebook posts. They present which types of errors commonly occur and co-occur and proceed to lay out a set of recommendations to reduce these types of errors. Generally speaking I think this type of data should be published and available, there is value here despite the relative simplicity of what it can contribute theoretically and practically. However, I have some major issues with the current write-up which you will find below.”

Answer 1: Thank you for your thoughtful review and valuable comments, which have helped improve the clarity and quality of the manuscript.

Comment 2: “First, I find the methodological detail lacking here. The authors state that "posts were collected from online spaces discussing opposition to vaccination or criticizing immunization policies" - given that the results show that there were no posts which do not contain statistical mis-reasoning - was post selection aimed exclusively at posts which argue against vaccination? This should be clearly stated. Further, the selection criteria seems not to have much systematicity to it - how was this done. Were all posts within a time period scraped - then criteria applied to retain those which adhere? Was an author simply scrolling and selecting based on the criteria but doing so "on the fly"?”

Answer 2: In response to another reviewer’s comment, a detailed clarification of the coding procedure was added, including how categories were defined, applied systematically across the dataset, and developed through an iterative process to ensure conceptual completeness without redundancy. In addition, the description of data collection was clarified to explicitly state that the sample was restricted to posts expressing opposition to vaccination, as well as to emphasise the purposive nature of the sampling and the systematic application of predefined inclusion and exclusion criteria.

Comment 3: “Second, it is unclear whether posts come from unique members or whether some members of these discussion groups contributed multiple posts? If posts come from unique posters then fine - however if the posts are not independent then the current analysis and the one I propose becomes problematic. E.g., if a single member posts twice but does not repeat the same mis-reasoning errors then there is an argument to be made that these should be combined in a single "argument" rather than separate "posts" as for that particular person, for their thought stream and argumentation there is co-occurrence of errors even if they do not occur within the same post.”

Answer 3: Thank you for this important and insightful comment. The analysis in this study was conducted at the level of individual posts. A limitation has been added to acknowledge that multiple posts may have originated from the same individual, including anonymous users whose identities could not be consistently traced. This limitation and its potential implications for the independence of observations are now explicitly discussed in the manuscript.

Comment 4: “Third, and the author touches on this as a limitation - what was the inductive approach to coding? Was it an established qualitative technique which then led to quantification after coding or was it not an established technique and protocol? For example, the posts could have been analysed initially by coding each statistical mis-reasoning error into an initial code - a very micro level where almost every code would be independent of each other - then in a second pass the micro-codes can be categorized into more broad categories - and then perhaps after a couple of passes of this the data would reveal what categories are present - whether there is redundancy/overlap and then pruning could be applied to end up with the categories. On the other hand it could have been pretty much a priori definition of categories before coding. It is unclear what was exactly done.”

Answer 4: In response to another reviewer’s comment, the description of the coding procedure was expanded to clarify how categories were defined and applied consistently across the dataset, how they were developed through an iterative, inductive process of identifying and refining patterns of misreasoning, and to present the final set of categories with definitions and illustrative examples in Table 1. The description has also been further clarified to explicitly outline the stepwise coding process, including the initial identification of individual instances of misreasoning and their subsequent iterative grouping and refinement into broader categories.

Comment 5: “Fourth, the statistical analysis seems arbitrary. Why are counts compared to 50%? No rationale is given for why 50% is a reasonable comparator. A % could be defined as practically i

Attachment Submitted filename: Point-by-point-response MARCH.2026.docx

# Peer review: Decision Letter 1

**DOI:** 10.1371/journal.pone.0355341.r003

**Authors:** José C. Perales

21 May 2026

PONE-D-25-65889R1

Statistical misreasoning in online content about vaccines: implications and recommendations for addressing disinformation

PLOS One

Dear Dr. Ordak,

Thank you for submitting your manuscript to PLOS ONE. After careful consideration, we feel that it has merit but does not fully meet PLOS ONE’s publication criteria as it currently stands. Therefore, we invite you to submit a revised version of the manuscript that addresses the points raised during the review process.

Both reviewers acknowledge significant improvements in your revision, though some concerns remain, primarily around the need for greater methodological clarity and transparency, and the importance of more explicitly acknowledging the limitations that cannot be addressed at this current stage.

Considering that, please submit your revised manuscript by Jul 05 2026 11:59PM. If you will need more time than this to complete your revisions, please reply to this message or contact the journal office at plosone@plos.org. When you're ready to submit your revision, log on to https://www.editorialmanager.com/pone/ and select the 'Submissions Needing Revision' folder to locate your manuscript file.

Please include the following items when submitting your revised manuscript:

- A letter that responds to each point raised by the academic editor and reviewer(s). You should upload this letter as a separate file labeled 'Response to Reviewers'.
- A marked-up copy of your manuscript that highlights changes made to the original version. You should upload this as a separate file labeled 'Revised Manuscript with Track Changes'.
- An unmarked version of your revised paper without tracked changes. You should upload this as a separate file labeled 'Manuscript'.

If you would like to make changes to your financial disclosure, please include your updated statement in your cover letter. Guidelines for resubmitting your figure files are available below the reviewer comments at the end of this letter.

If applicable, we recommend that you deposit your laboratory protocols in protocols.io to enhance the reproducibility of your results. Protocols.io assigns your protocol its own identifier (DOI) so that it can be cited independently in the future. For instructions see: https://journals.plos.org/plosone/s/submission-guidelines#loc-laboratory-protocols. Additionally, PLOS ONE offers an option for publishing peer-reviewed Lab Protocol articles, which describe protocols hosted on protocols.io. Read more information on sharing protocols at https://plos.org/protocols?utm_medium=editorial-email&utm_source=authorletters&utm_campaign=protocols.

As the corresponding author, your ORCID iD is verified in the submission system and will appear in the published article. PLOS supports the use of ORCID, and we encourage all coauthors to register for an ORCID iD and use it as well. Please encourage your coauthors to verify their ORCID iD within the submission system before final acceptance, as unverified ORCID iDs will not appear in the published article. *Only*  the individual author can complete the verification step; PLOS staff *cannot*  verify ORCID iDs on behalf of authors.

We look forward to receiving your revised manuscript.

Kind regards,

José C. Perales

Academic Editor

PLOS One

Journal Requirements:

If the reviewer comments include a recommendation to cite specific previously published works, please review and evaluate these publications to determine whether they are relevant and should be cited. There is no requirement to cite these works unless the editor has indicated otherwise.

[Note: HTML markup is below. Please do not edit.]

Reviewers' comments:

Reviewer's Responses to Questions

**Comments to the Author**

1. If the authors have adequately addressed your comments raised in a previous round of review and you feel that this manuscript is now acceptable for publication, you may indicate that here to bypass the “Comments to the Author” section, enter your conflict of interest statement in the “Confidential to Editor” section, and submit your "Accept" recommendation.

Reviewer #1: All comments have been addressed

Reviewer #2: (No Response)

**********

2. Is the manuscript technically sound, and do the data support the conclusions?

The manuscript must describe a technically sound piece of scientific research with data that supports the conclusions. Experiments must have been conducted rigorously, with appropriate controls, replication, and sample sizes. The conclusions must be drawn appropriately based on the data presented.

Reviewer #1: Yes

Reviewer #2: Yes

**********

3. Has the statistical analysis been performed appropriately and rigorously?

Reviewer #1: Yes

Reviewer #2: Yes

**********

4. Have the authors made all data underlying the findings in their manuscript fully available?

The PLOS Data policy requires authors to make all data underlying the findings described in their manuscript fully available without restriction, with rare exception (please refer to the Data Availability Statement in the manuscript PDF file). The data should be provided as part of the manuscript or its supporting information, or deposited to a public repository. For example, in addition to summary statistics, the data points behind means, medians and variance measures should be available. If there are restrictions on publicly sharing data—e.g. participant privacy or use of data from a third party—those must be specified.

Reviewer #1: Yes

Reviewer #2: No

**********

5. Is the manuscript presented in an intelligible fashion and written in standard English?

PLOS ONE does not copyedit accepted manuscripts, so the language in submitted articles must be clear, correct, and unambiguous. Any typographical or grammatical errors should be corrected at revision, so please note any specific errors here.

Reviewer #1: Yes

Reviewer #2: Yes

**********

6. Review Comments to the Author

Please use the space provided to explain your answers to the questions above. You may also include additional comments for the author, including concerns about dual publication, research ethics, or publication ethics. (Please upload your review as an attachment if it exceeds 20,000 characters)

Reviewer #1: I have read the revision of this manuscript. I think all my comments have been addressed.

The study has several problems and limitations that affect the generalization of the conclusions. But now that the limitations of the study are clearer, the reader will be able to judge the relevance of the results. If anything, the 50% threshold for the frequency analysis is still not well justified, so I would ask to include a brief explanation either in the Results or in the Discussion sections.

Reviewer #2: The detailed and comprehensive responses from the author are much appreciated. However, in light of comments from my fellow reviewer and the responses themselves, I have to say I still some issues with the current write-up. I am assigning this a "major revision" recommendation. I see the overall message as valuable, but at the same time what is here in methodological and other terms is quite limited and with the added core issues regarding the workflow - even if I would like more awareness of these biases through more publications - I cannot recommend publishing in this form.

Table 1 adds to the clarity but also clearly shows potential issues of categories not being well delineated.

For example. The "Cherry picking" descriptor states "selective use of numerical evidence (e.g., specific datasets, countries or time periods)...". But the example of denominator neglect includes reference to a specific time period so that example is a compound. Sure the emphasis is on "200 cases of complications" without taking into account the overall number of vaccinated individual but it is also aimed at a specific time period ("last month"). Likewise, small sample fallacy could be seen as cherry picking ("a few people I know" being cherry picked and the vast other information available being ignored). Perhaps I'm getting caught on the naming here - if it were "anecdotal personal experience" rather than "small sample fallacy" for this example then it would be better. One could argue that the example in cherry picking is also a compound of multiple biases. Yes the emphasis is on "one selected country" - but really the rest of it includes other biases. Linking the number of cases and number of vaccinations in a causal relationship for one, ignoring that testing is more frequent and in full swing at that point, that measures were relaxed, ignoring the make up of those new cases etc. Now, it may be the case that these were indeed counted as co-occurrences as only 8% of the posts had a single bias - but it's unclear whether this is the case or if coding issues produced these overlaps. There are other examples between the various categories.

I would also echo Reviewer 1 that it would make sense if additional raters were invited at this stage with the categories identified and given the posts to categorize and produce a measure of inter-rater reliability. Even with the edits, I do not find the explanation of the process very satisfying. Although it is more transparent - it does not increase my confidence in the reliability of the work.

See more minor issues below.

There are still places in the manuscript where clarity can be better. The phrase “statistically significant or of non-negligible magnitude were retained” but from what is written in text it seems the criterion was a p < 0.05 so the "or of non-negligible magnitude" should be removed, or if it was somehow taken into account - then a threshold should be introduced rather than the term "non-negligible".

Now that the 50% is explained as being the comparator for prevalence frequencies - it seems like a lot of fluff to simply state biases "X and Y occur in the majority of posts". The chi-squared tests seem like a tack-on which do not add much to the results or interpretations.

The author states in the discussion that co-occurrence may indicate "structural links" - however, it may simply be high salience - some biases are strong and prevalent, very salient - so these may be more prevalent and co-occur for that reason, not necessarily because there is a structural link (though there is a more theoretical discussion to be had on whether all biases are really at their root the same cognitive process etc.).

“Between 2023 and November 2025” - at least the month in 2023 should be in there to match how 2025 is reported - though if posts are not to be shared openly then there is no reason not to have exact dates in there.

There are two Table 1-s – the new table was added without adjusting numeration of the other tables.

I am still unconvinced by the data sharing statements. I would suggest pointing to exact TOS sections and perhaps having the "available upon reasonable request" type of statement in there if the data cannot be shared openly.

**********

7. PLOS authors have the option to publish the peer review history of their article (what does this mean?). If published, this will include your full peer review and any attached files.

If you choose “no”, your identity will remain anonymous but your review may still be made public.

**Do you want your identity to be public for this peer review?**  For information about this choice, including consent withdrawal, please see our Privacy Policy.

Reviewer #1: No

Reviewer #2: No

**********

[NOTE: If reviewer comments were submitted as an attachment file, they will be attached to this email and accessible via the submission site. Please log into your account, locate the manuscript record, and check for the action link "View Attachments". If this link does not appear, there are no attachment files.]

To ensure your figures meet our technical requirements, please review our figure guidelines: https://journals.plos.org/plosone/s/figures

You may also use PLOS’s free figure tool, NAAS, to help you prepare publication quality figures: https://journals.plos.org/plosone/s/figures#loc-tools-for-figure-preparation.

NAAS will assess whether your figures meet our technical requirements by comparing each figure against our figure specifications.

# Author comment: Author response to Decision Letter 2

**DOI:** 10.1371/journal.pone.0355341.r004

22 May 2026

Point-by-point response template

Date: 22.05.2026

Manuscript Number: PONE-D-25-65889R1

Title of Article: “Statistical misreasoning in online content about vaccines: implications and recommendations for addressing disinformation”

Name of the Corresponding Author: Michal Ordak

Email Address of the Corresponding Author: michal.ordak@wum.edu.pl

Dear Prof. Emily Chenette,

Thank you for sending the Reviewer’s comments regarding my paper entitled “Statistical misreasoning in online content about vaccines: implications and recommendations for addressing disinformation”, by M. Ordak (PONE-D-25-65889R1). I greatly appreciate all valuable suggestions proposed by the Reviewer, which I consider while preparing the revised version of the manuscript.

Specific Responses

Response to Reviewer #1:

Dear Reviewer nr 1,

Comment 1: “I have read the revision of this manuscript. I think all my comments have been addressed. The study has several problems and limitations that affect the generalization of the conclusions. But now that the limitations of the study are clearer, the reader will be able to judge the relevance of the results. If anything, the 50% threshold for the frequency analysis is still not well justified, so I would ask to include a brief explanation either in the Results or in the Discussion sections.”

Answer 1: Thank you for your positive evaluation of the revised manuscript and for acknowledging that the previous comments have been adequately addressed. In response to your remaining suggestion, I added a brief clarification in the Results section explaining that the 50% threshold was treated as a pragmatic indicator of majority occurrence within the analysed sample and was used to distinguish dominant from less prevalent forms of statistical misreasoning.

Response to Reviewer #2:

Dear Reviewer nr 2,

Comment 1: “The detailed and comprehensive responses from the author are much appreciated. However, in light of comments from my fellow reviewer and the responses themselves, I have to say I still some issues with the current write-up. I am assigning this a "major revision" recommendation. I see the overall message as valuable, but at the same time what is here in methodological and other terms is quite limited and with the added core issues regarding the workflow - even if I would like more awareness of these biases through more publications - I cannot recommend publishing in this form.”

Answer 1: Thank you for your detailed evaluation of the manuscript and for acknowledging the value and relevance of the overall message of the study. I also appreciate your additional comments and methodological suggestions, which once again contributed to improving the clarity and quality of the manuscript. All remarks were carefully considered and addressed in the revised version.

Comment 2: “Table 1 adds to the clarity but also clearly shows potential issues of categories not being well delineated.

For example. The "Cherry picking" descriptor states "selective use of numerical evidence (e.g., specific datasets, countries or time periods)...". But the example of denominator neglect includes reference to a specific time period so that example is a compound. Sure the emphasis is on "200 cases of complications" without taking into account the overall number of vaccinated individual but it is also aimed at a specific time period ("last month"). Likewise, small sample fallacy could be seen as cherry picking ("a few people I know" being cherry picked and the vast other information available being ignored). Perhaps I'm getting caught on the naming here - if it were "anecdotal personal experience" rather than "small sample fallacy" for this example then it would be better. One could argue that the example in cherry picking is also a compound of multiple biases. Yes the emphasis is on "one selected country" - but really the rest of it includes other biases. Linking the number of cases and number of vaccinations in a causal relationship for one, ignoring that testing is more frequent and in full swing at that point, that measures were relaxed, ignoring the make up of those new cases etc. Now, it may be the case that these were indeed counted as co-occurrences as only 8% of the posts had a single bias - but it's unclear whether this is the case or if coding issues produced these overlaps. There are other examples between the various categories.”

Answer 2: Thank you for this helpful observation. To clarify the intended relationship between categories, the manuscript was revised to explicitly state that the coding categories were not treated as fully mutually exclusive, as individual posts frequently contained overlapping forms of statistical misreasoning. In addition, several formulations emphasizing “non-redundancy” and avoidance of overlap were replaced with wording focused on “analytical distinctiveness”, “analytically differentiated typology” and minimizing “conceptual redundancy” in order to better reflect the analytical purpose of the framework and the compound nature of many examples presented in Table 1.

Comment 3: “I would also echo Reviewer 1 that it would make sense if additional raters were invited at this stage with the categories identified and given the posts to categorize and produce a measure of inter-rater reliability. Even with the edits, I do not find the explanation of the process very satisfying. Although it is more transparent - it does not increase my confidence in the reliability of the work.”

Answer 3: Thank you for this important observation. In response, the limitations section was expanded to more explicitly acknowledge that the absence of independent coding prevents formal assessment of the reproducibility and reliability of classification decisions. The revised manuscript now clarifies that the proposed framework should be regarded as exploratory and theory-generating rather than as a fully validated and independently reproducible coding system, and it explicitly identifies independent coding procedures and formal inter-rater reliability assessment as important directions for future validation of the framework.

Comment 4: “There are still places in the manuscript where clarity can be better. The phrase “statistically significant or of non-negligible magnitude were retained” but from what is written in text it seems the criterion was a p < 0.05 so the "or of non-negligible magnitude" should be removed, or if it was somehow taken into account - then a threshold should be introduced rather than the term "non-negligible.”

Answer 4: Thank you for this comment. In response, I revised the relevant sentence in the Results section by removing the reference to “non-negligible magnitude” so that the interpretation criteria are fully consistent with the statistical significance threshold (p < 0.05) described in the manuscript.

Comment 5: “Now that the 50% is explained as being the comparator for prevalence frequencies - it seems like a lot of fluff to simply state biases "X and Y occur in the majority of posts". The chi-squared tests seem like a tack-on which do not add much to the results or interpretations.”

Answer 5: Thank you for this valuable comment. In response, I revised the wording in the Results section to reduce the emphasis placed on the chi-square tests and to better reflect the primarily descriptive nature of the analysis. In addition, following the suggestion of the first reviewer, I added a clarifying sentence explaining that the 50% threshold was treated as a pragmatic indicator of majority occurrence within the analysed sample and was used to distinguish dominant from less prevalent forms of statistical misreasoning.

Comment 6: “The author states in the discussion that co-occurrence may indicate "structural links" - however, it may simply be high salience - some biases are strong and prevalent, very salient - so these may be more prevalent and co-occur for that reason, not necessarily because there is a structural link (though there is a more theoretical discussion to be had on whether all biases are really at their root the same cognitive process etc.).”

Answer 6: I revised the discussion section to avoid overly strong interpretations of co-occurrence patterns as evidence of structural links between biases and clarified that the observed co-occurrences may also reflect the high prevalence and salience of particular forms of statistical misreasoning.

Comment 7: “Between 2023 and November 2025” - at least the month in 2023 should be in there to match how 2025 is reported - though if posts are not to be shared openly then there is no reason not to have exact dates in there.”

Answer 7: I clarified the timeframe of data collection by specifying both the starting and ending months of the analysed period.

Comment 8: “There are two Table 1-s – the new table was added without adjusting numeration of the other tables.”

Answer 8: Thank you for this valid comment. In response, the table numbering has been corrected.

Comment 9: “I am still unconvinced by the data sharing statements. I would suggest pointing to exact TOS sections and perhaps having the "available upon reasonable request" type of statement in there if the data cannot be shared openly.”

Answer 9: I revised the data availability statement to clarify that the original Facebook posts are not publicly shared due to privacy considerations and restrictions related to Facebook Terms of Service governing the redistribution of user-generated content.

Attachment Submitted filename: Point-by-point-response MAY.2026.docx

# Peer review: Decision Letter 2

**DOI:** 10.1371/journal.pone.0355341.r005

**Authors:** José C. Perales

17 Jul 2026

<div>PONE-D-25-65889R2

Statistical misreasoning in online content about vaccines: implications and recommendations for addressing disinformation

PLOS One

Dear Dr. Ordak,

Thank you for submitting your revised manuscript to PLOS ONE.

As you can see, the two reviewers are now more positive about the prospects of your manuscript, and mention both minor stylistic issues and more substantial methodological problems that, while not addressable at this point, have at least been sufficiently acknowledged to avoid a misreading of the results. Please correct the former and consider the possibility of further elaborating on the latter. If these changes are diligently made, no further reviews will be required for me to make a definitive decision.

Please submit your revised manuscript by Aug 31 2026 11:59PM. If you will need more time than this to complete your revisions, please reply to this message or contact the journal office at plosone@plos.org. When you're ready to submit your revision, log on to https://www.editorialmanager.com/pone/ and select the 'Submissions Needing Revision' folder to locate your manuscript file.

Please include the following items when submitting your revised manuscript:

- A letter that responds to each point raised by the academic editor and reviewer(s). You should upload this letter as a separate file labeled 'Response to Reviewers'.
- A marked-up copy of your manuscript that highlights changes made to the original version. You should upload this as a separate file labeled 'Revised Manuscript with Track Changes'.
- An unmarked version of your revised paper without tracked changes. You should upload this as a separate file labeled 'Manuscript'.

If you would like to make changes to your financial disclosure, please include your updated statement in your cover letter. Guidelines for resubmitting your figure files are available below the reviewer comments at the end of this letter.

If applicable, we recommend that you deposit your laboratory protocols in protocols.io to enhance the reproducibility of your results. Protocols.io assigns your protocol its own identifier (DOI) so that it can be cited independently in the future. For instructions see: https://journals.plos.org/plosone/s/submission-guidelines#loc-laboratory-protocols. Additionally, PLOS ONE offers an option for publishing peer-reviewed Lab Protocol articles, which describe protocols hosted on protocols.io. Read more information on sharing protocols at https://plos.org/protocols?utm_medium=editorial-email&utm_source=authorletters&utm_campaign=protocols.

As the corresponding author, your ORCID iD is verified in the submission system and will appear in the published article. PLOS supports the use of ORCID, and we encourage all coauthors to register for an ORCID iD and use it as well. Please encourage your coauthors to verify their ORCID iD within the submission system before final acceptance, as unverified ORCID iDs will not appear in the published article. *Only*  the individual author can complete the verification step; PLOS staff *cannot*  verify ORCID iDs on behalf of authors.

We look forward to receiving your revised manuscript.

Kind regards,

José C. Perales

Academic Editor

PLOS One

Journal Requirements:

1. If the reviewer comments include a recommendation to cite specific previously published works, please review and evaluate these publications to determine whether they are relevant and should be cited. There is no requirement to cite these works unless the editor has indicated otherwise.

2. Please review your reference list to ensure that it is complete and correct. If you have cited papers that have been retracted, please include the rationale for doing so in the manuscript text, or remove these references and replace them with relevant current references. Any changes to the reference list should be mentioned in the rebuttal letter that accompanies your revised manuscript. If you need to cite a retracted article, indicate the article’s retracted status in the References list and also include a citation and full reference for the retraction notice.

[Note: HTML markup is below. Please do not edit.]

Reviewers' comments:

Reviewer's Responses to Questions

**Comments to the Author**

1. If the authors have adequately addressed your comments raised in a previous round of review and you feel that this manuscript is now acceptable for publication, you may indicate that here to bypass the “Comments to the Author” section, enter your conflict of interest statement in the “Confidential to Editor” section, and submit your "Accept" recommendation.

Reviewer #1: All comments have been addressed

Reviewer #2: All comments have been addressed

**********

2. Is the manuscript technically sound, and do the data support the conclusions?

The manuscript must describe a technically sound piece of scientific research with data that supports the conclusions. Experiments must have been conducted rigorously, with appropriate controls, replication, and sample sizes. The conclusions must be drawn appropriately based on the data presented.

Reviewer #1: Yes

Reviewer #2: Yes

**********

3. Has the statistical analysis been performed appropriately and rigorously?

Reviewer #1: Yes

Reviewer #2: Yes

**********

4. Have the authors made all data underlying the findings in their manuscript fully available?

The PLOS Data policy requires authors to make all data underlying the findings described in their manuscript fully available without restriction, with rare exception (please refer to the Data Availability Statement in the manuscript PDF file). The data should be provided as part of the manuscript or its supporting information, or deposited to a public repository. For example, in addition to summary statistics, the data points behind means, medians and variance measures should be available. If there are restrictions on publicly sharing data—e.g. participant privacy or use of data from a third party—those must be specified.

Reviewer #1: No

Reviewer #2: No

**********

5. Is the manuscript presented in an intelligible fashion and written in standard English?

PLOS ONE does not copyedit accepted manuscripts, so the language in submitted articles must be clear, correct, and unambiguous. Any typographical or grammatical errors should be corrected at revision, so please note any specific errors here.

Reviewer #1: Yes

Reviewer #2: Yes

**********

6. Review Comments to the Author

Please use the space provided to explain your answers to the questions above. You may also include additional comments for the author, including concerns about dual publication, research ethics, or publication ethics. (Please upload your review as an attachment if it exceeds 20,000 characters)

Reviewer #1: I have read the revised version of the manuscript.

All of my previous comments have been adequately addressed. The only remaining issue was the justification for the 50% threshold, which is now included. While I do not think the "pragmatic" rationale is entirely convincing, I believe it is acceptable as long as it is clearly and transparently reported.

Overall, although the study has several limitations (some of which were appropriately highlighted by the other reviewer), I think these limitations are adequately acknowledged and discussed, so readers are unlikely to be misled.

Minor comment: I noticed some inconsistencies in the use of decimal notation (e.g., 0.001 vs. 0,001). Please ensure that the notation is consistent throughout the manuscript.

Reviewer #2: I appreciate the author's responses. I still feel that the chi-squared tests versus a 50% thresholds are not all that informative but they also don't really hurt the paper (except for making it longer). A minor formatting issue is that some p values in the table have decimal commas, rather than dots so just general polish of these types of things to make it cleaner is probably necessary.

Considering that the main methodological issues cannot be solved without additional workload and that the author addresses this as an issue in the discussion, I am leaving it to the editor to decide whether Table 1 and the methodological weaknesses require more work or if addressing these points as they have been is enough.

Therefore I'm recommending "minor revisions" but if the editor decides that additional work is not required I do not expect to get this back on my proverbial desk and in that case consider the recommendation as "accept".

**********

7. PLOS authors have the option to publish the peer review history of their article (what does this mean?). If published, this will include your full peer review and any attached files.

If you choose “no”, your identity will remain anonymous but your review may still be made public.

**Do you want your identity to be public for this peer review?**  For information about this choice, including consent withdrawal, please see our Privacy Policy.

Reviewer #1: No

Reviewer #2: No

**********

[NOTE: If reviewer comments were submitted as an attachment file, they will be attached to this email and accessible via the submission site. Please log into your account, locate the manuscript record, and check for the action link "View Attachments". If this link does not appear, there are no attachment files.]

To ensure your figures meet our technical requirements, please review our figure guidelines: https://journals.plos.org/plosone/s/figures

You may also use PLOS’s free figure tool, NAAS, to help you prepare publication quality figures: https://journals.plos.org/plosone/s/figures#loc-tools-for-figure-preparation.

NAAS will assess whether your figures meet our technical requirements by comparing each figure against our figure specifications.

# Author comment: Author response to Decision Letter 3

**DOI:** 10.1371/journal.pone.0355341.r006

18 Jul 2026

Point-by-point response template

Date: 18.07.2026

Manuscript Number: PONE-D-25-65889R2

Title of Article: “Statistical misreasoning in online content about vaccines: implications and recommendations for addressing disinformation”

Name of the Corresponding Author: Michal Ordak

Email Address of the Corresponding Author: michal.ordak@wum.edu.pl

Dear Prof. Emily Chenette,

Thank you for sending the Reviewer’s comments regarding my paper entitled “Statistical misreasoning in online content about vaccines: implications and recommendations for addressing disinformation”, by M. Ordak (PONE-D-25-65889R2). I greatly appreciate all valuable suggestions proposed by the Reviewer, which I consider while preparing the revised version of the manuscript.

Specific Responses

Response to Reviewer #1:

Dear Reviewer nr 1,

Comment 1: “I have read the revised version of the manuscript.

All of my previous comments have been adequately addressed. The only remaining issue was the justification for the 50% threshold, which is now included. While I do not think the "pragmatic" rationale is entirely convincing, I believe it is acceptable as long as it is clearly and transparently reported. Overall, although the study has several limitations (some of which were appropriately highlighted by the other reviewer), I think these limitations are adequately acknowledged and discussed, so readers are unlikely to be misled.

Minor comment: I noticed some inconsistencies in the use of decimal notation (e.g., 0.001 vs. 0,001). Please ensure that the notation is consistent throughout the manuscript.”

Answer 1: I thank the Reviewer for the positive assessment and for confirming that my revisions have adequately addressed the previous comments. I appreciate the Reviewer's thoughtful evaluation and positive overall assessment of the manuscript. Thank you for pointing this out. I have corrected the decimal notation in the p values throughout the manuscript to ensure consistency.

Response to Reviewer #2:

Dear Reviewer nr 2,

Comment 1: “I appreciate the author's responses. I still feel that the chi-squared tests versus a 50% thresholds are not all that informative but they also don't really hurt the paper (except for making it longer). A minor formatting issue is that some p values in the table have decimal commas, rather than dots so just general polish of these types of things to make it cleaner is probably necessary. Considering that the main methodological issues cannot be solved without additional workload and that the author addresses this as an issue in the discussion, I am leaving it to the editor to decide whether Table 1 and the methodological weaknesses require more work or if addressing these points as they have been is enough. Therefore I'm recommending "minor revisions" but if the editor decides that additional work is not required I do not expect to get this back on my proverbial desk and in that case consider the recommendation as "accept".”

Answer 1: Thank you for your positive evaluation of the manuscript and for your constructive comments. Thank you for acknowledging that the main methodological limitations have been addressed in the discussion. In accordance with your comment, I have revised the formatting of the p values throughout the manuscript by replacing decimal commas with decimal points to ensure consistent notation.

Attachment Submitted filename: Point-by-point-response July 2026.docx

# Editor report: Decision Letter 3

**DOI:** 10.1371/journal.pone.0355341.r007

**Authors:** José C. Perales

20 Jul 2026

Statistical misreasoning in online content about vaccines: implications and recommendations for addressing disinformation

PONE-D-25-65889R3

Dear Dr. Ordak,

We’re pleased to inform you that your manuscript has been judged scientifically suitable for publication and will be formally accepted for publication once it meets all outstanding technical requirements.

Within one week, you’ll receive an e-mail detailing the required amendments. When these have been addressed, you’ll receive a formal acceptance letter and your manuscript will be scheduled for publication.

An invoice will be generated when your article is formally accepted. Please note, if your institution has a publishing partnership with PLOS and your article meets the relevant criteria, all or part of your publication costs will be covered. Please make sure your user information is up-to-date by logging into Editorial Manager at Editorial Manager® and clicking the ‘Update My Information' link at the top of the page. For questions related to billing, please contact billing support.

If your institution or institutions have a press office, please notify them about your upcoming paper to help maximize its impact. If they’ll be preparing press materials, please inform our press team as soon as possible -- no later than 48 hours after receiving the formal acceptance. Your manuscript will remain under strict press embargo until 2 pm Eastern Time on the date of publication. For more information, please contact onepress@plos.org.

Kind regards,

José C. Perales

Academic Editor

PLOS One

Additional Editor Comments (optional):

Reviewers' comments:

# Editor report: Acceptance letter

**DOI:** 10.1371/journal.pone.0355341.r008

**Authors:** José C. Perales

PONE-D-25-65889R3

PLOS One

Dear Dr. Ordak,

I'm pleased to inform you that your manuscript has been deemed suitable for publication in PLOS One. Congratulations! Your manuscript is now being handed over to our production team.

At this stage, our production department will prepare your paper for publication. This includes ensuring the following:

* All references, tables, and figures are properly cited

* All relevant supporting information is included in the manuscript submission,

* There are no issues that prevent the paper from being properly typeset

You will receive further instructions from the production team, including instructions on how to review your proof when it is ready. Please keep in mind that we are working through a large volume of accepted articles, so please give us a few days to review your paper and let you know the next and final steps.

Lastly, if your institution or institutions have a press office, please let them know about your upcoming paper now to help maximize its impact. If they'll be preparing press materials, please inform our press team within the next 48 hours. Your manuscript will remain under strict press embargo until 2 pm Eastern Time on the date of publication. For more information, please contact onepress@plos.org.

You will receive an invoice from PLOS for your publication fee after your manuscript has reached the completed accept phase. If you receive an email requesting payment before acceptance or for any other service, this may be a phishing scheme. Learn how to identify phishing emails and protect your accounts at https://explore.plos.org/phishing.

If we can help with anything else, please email us at customercare@plos.org.

Thank you for submitting your work to PLOS ONE and supporting open access.

Kind regards,

PLOS ONE Editorial Office Staff

on behalf of

Dr. José C. Perales

Academic Editor

PLOS One
